# Potential biomarkers for multiple sclerosis stage from targeted proteomics and microRNA sequencing

**DOI:** 10.1093/braincomms/fcae209

**Published:** 2024-06-13

**Authors:** Ineke L Tan, Rutger Modderman, Anna Stachurska, Rodrigo Almeida, Riemer de Vries, Dorothea J Heersema, Ranko Gacesa, Cisca Wijmenga, Iris H Jonkers, Jan F Meilof, Sebo Withoff

**Affiliations:** Department of Genetics, University of Groningen, University Medical Center Groningen, 9713 GZ Groningen, The Netherlands; Department of Gastroenterology and Hepatology, University of Groningen, University Medical Center Groningen, 9713 GZ Groningen, The Netherlands; Department of Genetics, University of Groningen, University Medical Center Groningen, 9713 GZ Groningen, The Netherlands; Department of Genetics, University of Groningen, University Medical Center Groningen, 9713 GZ Groningen, The Netherlands; Telespazio Belgium S.R.L. for the European Space Agency (ESA), 2200AG Noordwijk, The Netherlands; Department of Neurology, University of Groningen, University Medical Center Groningen, 9713 GZ Groningen, The Netherlands; Department of Neurology, University of Groningen, University Medical Center Groningen, 9713 GZ Groningen, The Netherlands; MS Center Noord Nederland, University of Groningen, University Medical Center Groningen, Groningen, The Netherlands; Department of Genetics, University of Groningen, University Medical Center Groningen, 9713 GZ Groningen, The Netherlands; Department of Gastroenterology and Hepatology, University of Groningen, University Medical Center Groningen, 9713 GZ Groningen, The Netherlands; Department of Genetics, University of Groningen, University Medical Center Groningen, 9713 GZ Groningen, The Netherlands; Department of Genetics, University of Groningen, University Medical Center Groningen, 9713 GZ Groningen, The Netherlands; MS Center Noord Nederland, University of Groningen, University Medical Center Groningen, Groningen, The Netherlands; Department of Biomedical Sciences of Cells and Systems, University Medical Center Groningen, 9713 GZ Groningen, The Netherlands; Department of Genetics, University of Groningen, University Medical Center Groningen, 9713 GZ Groningen, The Netherlands

**Keywords:** multiple sclerosis, biomarker, disease stage, microRNA sequencing, proteomics

## Abstract

Multiple sclerosis is a chronic demyelinating disease of the central nervous system. There is a need for new circulating biomarkers for multiple sclerosis, in particular, markers that differentiate multiple sclerosis subtypes (relapsing–remitting, secondary progressive and primary progressive multiple sclerosis), as this can help in making treatment decisions. In this study, we explore two classes of potential multiple sclerosis biomarkers—proteins and microRNAs—circulating in the cerebrospinal fluid and serum. Targeted medium-throughput proteomics (92 proteins) and microRNA sequencing were performed on serum samples collected in a cross-sectional case–control cohort (cohort I, controls *n* = 30, multiple sclerosis *n* = 75) and a prospective multiple sclerosis cohort (cohort II, *n* = 93). For cohort I, we also made these measurements in paired cerebrospinal fluid samples. In the cohort I cerebrospinal fluid, we observed differences between multiple sclerosis and controls for 13 proteins, including some previously described to be markers for multiple sclerosis [e.g. CD27, C-X-C motif chemokine 13 (CXCL13) and interleukin-7 (IL7)]. No microRNAs were significantly differentially expressed between multiple sclerosis and controls in the cerebrospinal fluid. In serum, 10 proteins, including angiopoietin-1 receptor (TIE2), and 16 microRNAs were significantly different between relapsing–remitting multiple sclerosis and secondary progressive multiple sclerosis after performing a meta-analysis combining both cohorts. In the prospective part of the study, participants with relapsing–remitting multiple sclerosis were followed for around 3 years, during which time 12 participants converted to secondary progressive multiple sclerosis. In these longitudinally collected serum samples, we observed a peak in granzyme B, A and H proteins around the time of conversion. Single-sample enrichment analysis of serum microRNA profiles revealed that the peak in granzyme B levels around conversion coincides with enrichment for microRNAs that are enriched in CD4+, CD8+ and natural killer cells (e.g. miRNA-150). We identified several proteins and microRNAs in serum that represent potential biomarkers for relapsing–remitting and secondary progressive multiple sclerosis. Conversion to secondary progressive disease is marked by a peak in granzyme B levels and enrichment for immune-related microRNAs. This indicates that specific immune cell-driven processes may contribute to the conversion of relapsing–remitting multiple sclerosis to secondary progressive multiple sclerosis.

## Teaser

Tan *et al*. describe protein and microRNA biomarkers associated with disease progression in multiple sclerosis. A peak in circulating granzyme B levels was observed around the conversion from relapsing–remitting to secondary progressive multiple sclerosis. These findings provide mechanistic insights into disease state and progression, potentially aiding in developing future targeted therapies.

## Graphical Abstract

**Graphical Abstract:**
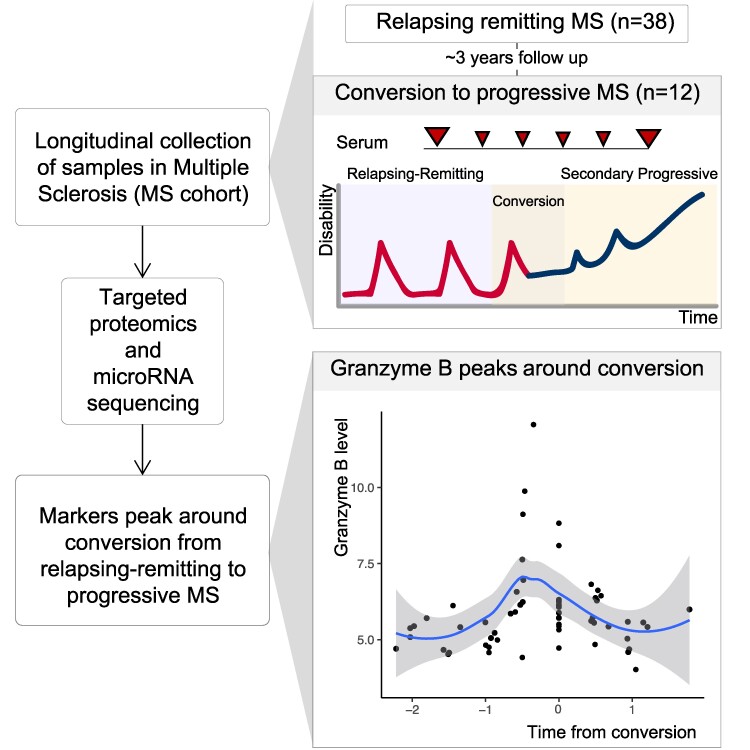

## Introduction

Multiple sclerosis is a chronic disease of the central nervous system that is characterized by inflammation-driven demyelination and progressive axonal and neuronal damage.^1,2^ The most recent genetic association study on multiple sclerosis reported more than 200 genetic susceptibility variants, which mainly affect the expression of immune-related genes. It is thought that deregulation of these gene products contributes to multiple sclerosis aetiology by deregulating multiple pathways involved in the peripheral and central (e.g. microglia) immune cells of both the innate and adaptive immune systems.^3,4^

In around 85% of multiple sclerosis patients, the disease course starts as relapsing–remitting multiple sclerosis, in which relapses of inflammatory demyelination occur alternating with episodes of stable neurological features.^5,6^ In most of these patients, relapse-independent neurological disability starts to manifest within two decades after disease diagnosis. This process is referred to as the secondary progressive phase of multiple sclerosis.^5,7^ The remaining 15% of multiple sclerosis patients are classified as primary progressive multiple sclerosis patients at first diagnosis. These patients present with a progressive disease phenotype from disease onset, with no preceding relapsing–remitting phase detected.^5^

Currently, multiple sclerosis diagnosis is based on the presence of (recurring) clinical symptoms and disease-typical magnetic resonance imaging abnormalities of the brain and spinal cord and on the detection of oligoclonal immunoglobulin bands in CSF.^6^ Although this diagnosis can be reliably established, there are no biomarkers that predict the rate of disability progression or signal when a patient has developed secondary progressive multiple sclerosis. Currently, the conversion of relapsing–remitting multiple sclerosis to secondary progressive multiple sclerosis can only be defined retrospectively based on increased neurological symptoms and supported by magnetic resonance imaging data indicating the accumulation of irreversible brain damage, independent of inflammatory relapses. The correct classification of a progressive disease in a patient with multiple sclerosis is thus often delayed for months or years.^8^ This is a shortcoming because most approved multiple sclerosis treatments target the inflammatory component of multiple sclerosis and have been approved specifically for relapsing–remitting multiple sclerosis.^2^ These drugs show limited effectiveness in progressive multiple sclerosis, as immunomodulatory drugs cannot sufficiently inhibit the neurodegenerative processes that lead to disability.^2,5^ As these drugs have substantial side-effects and only limited effectiveness in progressive multiple sclerosis, it would be beneficial for patients to identify biomarkers specific to disease stage, which would enable the timely cessation of immunomodulatory treatment. Unfortunately, there are currently no biomarkers that detect the conversion from relapsing–remitting to secondary progressive multiple sclerosis.

In the search for novel biomarkers for the different subtypes of multiple sclerosis that are measurable in more easily accessible biofluids, such as CSF or serum, several potential biomarker sources have gained attention. Targeted medium-throughput proteomics technologies, such as the multiplex proximity extension assays, provide attractive assays for biomarker discovery as they can quantify multiple proteins simultaneously in low sample volumes (1 µL).^9^ Another class of molecular biomarkers is extracellular microRNAs (miRNAs), which are small (∼20 nucleotides) non-coding RNAs circulating in blood and altered miRNA profiles having been associated with immune diseases and even with immune disease stage.^10-14^

In the current study, we prioritize inflammatory proteins and circulating miRNAs as potential biomarkers in CSF or serum that could help to better discriminate relapsing–remitting multiple sclerosis from the multiple sclerosis subtypes with a predominantly neurodegenerative component (secondary progressive multiple sclerosis and primary progressive multiple sclerosis). For this purpose, we applied protein arrays and miRNA sequencing in a large cross-sectional cell-free serum and CSF sample collection (obtained from the Dutch Brain Bank) and in longitudinal samples of a well-documented cohort of patients with multiple sclerosis who were seen and treated at the University Medical Center Groningen. We identify inflammatory signatures in blood that peak around the conversion from the relapsing–remitting phase to the secondary progressive phase of disease, which suggest that immune cell-driven events play a role in conversion.

## Materials and methods

### Sample collection

#### Cohort I: cross-sectional case–control cohort

**Figure 1:**
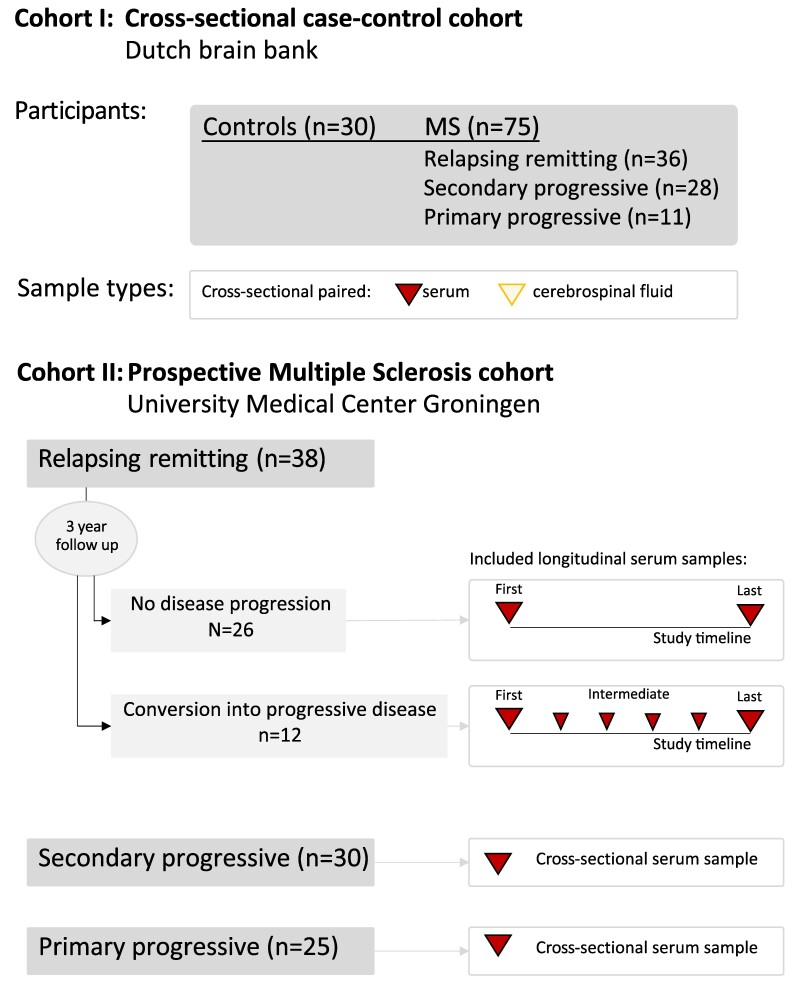
**Overview sample selection for protein and miRNA analyses.** In cohort I, paired serum and cerebrospinal fluid samples were collected cross-sectionally from controls (non-inflammatory neurological controls) and multiple sclerosis patients. In cohort II, serum samples were collected prospectively. For the relapsing–remitting patients in cohort II, samples were collected at every outpatient clinic visit during a follow-up of ∼3 years. For individuals who did not show disease progression, the first and last samples were included. For individuals who showed conversion to progressive disease, intermediate samples were included in the analyses, as well. In cohort II, cross-sectional samples were collected for patients with progressive disease.

Cross-sectional, paired cell-free serum and cell-free CSF samples were collected in the context of a previous study^15^ and stored in the Dutch Brain Bank (https://www.brainbank.nl). Participants from four categories were included: relapsing–remitting multiple sclerosis, secondary progressive multiple sclerosis, primary progressive multiple sclerosis and controls [individuals with other (non-inflammatory) neurological diseases] (see Fig. 1, cohort I). Paired CSF and serum samples were collected cross-sectionally. For the current study, we included only participants who did not use disease-modifying drugs at the time of inclusion. Supplementary Table 1 provides an overview of the cohort I samples analysed in this study and their clinical characteristics.

#### Cohort II: prospective multiple sclerosis cohort

Serum samples collected in the prospective multiple sclerosis cohort were used to identify biomarkers specific to disease subtype and to validate biomarkers longitudinally. Samples were collected from multiple sclerosis patients between 2014 and 2018 (see Fig. 1), and the cell-free serum was stored in the University Medical Center Groningen.

We included 38 out of the 61 patients with relapsing–remitting multiple sclerosis at inclusion in the current study. Blood was drawn from these patients at each regular doctor's visit (approx. every 6 months). During the follow-up, 12/61 patients converted from the relapsing–remitting phase to the secondary progressive phase of multiple sclerosis (‘converters’). For these converters, we analysed additional intermediate samples (see Fig. 1). For the remaining patients with relapsing–remitting multiple sclerosis who did not progress to secondary progressive multiple sclerosis during the study, we included 26 individuals selected based on age (to match the age of the converters) and analysed the first and available samples. Additionally, we collected blood from secondary progressive multiple sclerosis and primary progressive multiple sclerosis patients one time. All patients were diagnosed with clinically defined multiple sclerosis according to the revised McDonald criteria.^6,16^ Diagnosis of secondary progressive multiple sclerosis was confirmed by the treating physician (DJH) and at least one independent neurologist (RdV, JFM) according to the Lublin criteria.^7,17,18^  Supplementary Table 1 provides an overview of the cohort II samples analysed in this study and the clinical characteristics of the patients, including an overview of disease-modifying treatments.

Both the case–control study and the longitudinal study were performed in line with protocols approved by the local ethical review boards. All participants gave written informed consent.

#### Targeted proteomics with multiplex proximity extension assays

Protein levels in CSF (cohort I) and serum samples (cohorts I and II) were measured using the Olink Proseek multiplex immuno-oncology panel (Olink Bioscience, Uppsala, Sweden), which allows for a simultaneous evaluation of 92 proteins in 1 µL of sample (see Supplementary Table 2 for an overview of the proteins analysed). All samples were randomized before filling out plates to minimize differences in technical bias between groups (e.g. multiple sclerosis and controls) and sample types (CSF and serum). The readout of the proximity extension assays technology is Ct values derived from a qPCR reaction. These Ct values are subsequently translated into a measure of protein level in the sample, NPX values (log2 scale), which are normalized for the interplate control values. Quality control (QC) was performed according to the standard criteria provided by Olink^9^ (https://www.olink.com/content/uploads/2021/09/olink-data-normalization-white-paper-v2.0.pdf). In the QC step of the protein profiling experiments, three serum samples had to be excluded from cohort I due to low quality. All cohort II samples passed QC. Protein values below the limit of detection (LOD) were replaced by LOD/square root (2). Only the proteins with values above the LOD in >25% of the samples were considered for further analyses (https://www.olink.com/content/uploads/2021/09/olink-data-normalization-white-paper-v2.0.pdf).

After profiling cohort I and before profiling cohort II, the multiplex protein panel was updated (from product number 95311 to product number 95310), with the updated panel incorporating new probes for some of the proteins detected. In this update, the probes for tumour necrosis factor (TNF-)-alfa, interferon (IFN-)-gamma and CD8 were updated; the probes for interleukin (IL-)-21, IL-35, vascular endothelial growth factor C and IFN-beta were removed; and probes for IL-15, killer cell immunoglobulin-like receptor 3DL1, lymphocyte activation gene 3 protein and mucin-16 were added. Despite these changes, the analytical range remained the same for the 85 analytes that overlapped between the two panels. Supplementary Table 2 gives an overview of all the proteins measured in the two runs, including detection rate and analytical range. To further ensure comparable results across the two runs, 11 ‘bridging’ samples from the first cohort were reanalysed using the new panel, and we observed a high correlation between the two runs (see Supplementary Fig. 1A). For the 71 proteins with a high correlation coefficient > 0.7 in the bridging samples (Supplementary Fig. 1B), the NPX values of all samples in the second run were corrected using a correction accounting for the difference in the bridging samples between runs, per protein (Supplementary Fig. 1C).

### RNA isolation, small-RNA library preparation and sequencing and alignment of miRNA reads

After isolating extracellular RNA from serum and CSF samples, small-RNA libraries were prepared and sequenced according to the methods described in Tan *et al*.^19^ All RNA isolations, library preparations and sequencing (Illumina HiSeq2500) were performed in the University Medical Center Groningen. Raw reads were then aligned to the reference database miRBase 22.^19^

### Alignment of miRNA reads and QC of the miRNA profiles

For the serum samples, miRNA libraries of more than 100 different aligned miRNAs with >1 count (minimal cut-off for library diversity) and more than 1000 miRNA aligned counts in total (minimal cut-off for library size) were subjected to further QC steps (see Supplementary Methods and Supplementary Figs 9–11 for a more detailed overview). For the CSF samples, a quality cut-off of 50 different miRNAs was used for the library diversity because the miRNA profiles of CSF samples are known to be less diverse than serum libraries (see Supplementary Methods and Supplementary Fig. 9). In cohort I (cross-sectional case–control cohort), 75/98 serum miRNA libraries and 65/91 CSF miRNA libraries passed QC. In cohort II (prospective multiple sclerosis cohort), 137/140 miRNA libraries passed the QC steps.

## Statistical analyses

All statistical analyses were performed in R (version 3.6.2). All figures were generated using the R-package ggplot2 (version 3.1.0, https://ggplot2.tidyverse.org). We used the R-package compareGroups (version 4.0.0, https://cran.r-project.org/web/packages/compareGroups/index.html) to assess differences in the baseline characteristics (see Supplementary Methods for more detailed information).

We tested whether the 13 proteins in CSF that were different between multiple sclerosis and controls can distinguish multiple sclerosis from controls using the multivariate generalized linear models described in detail in the Supplementary Methods.

A differential expression analysis of the miRNA sequencing data was performed using the DESeq2 package using batch, age and sex as covariates (version 1.22.2, https://github.com/mikelove/DESeq2). See the Supplementary Methods for more detailed information on the differential expression analysis. The *P*-values for proteins and miRNAs were corrected for the number of analytes tested according to Benjamini–Hochberg [adjusted *P*-value (FDR)].^20^ MiRNAs were considered significantly differentially expressed at an FDR-corrected *P*-value < 0.1. All statistical analyses for the miRNA data were performed for both cohorts separately, as no bridging samples from cohort I were re-measured while measuring cohort II.

To assess the consistency of the subgroup differences between relapsing–remitting multiple sclerosis and secondary progressive multiple sclerosis across the two separate cohorts, the results for the markers with a nominal *P*-value < 0.1 in the analyses in the separate cohorts were combined in a meta-analysis, as outlined in the Supplementary Methods.

To search for associations of markers with disease progression in the converters, we associated marker levels with a variable, called ‘Time from conversion’, which is the time between the taking of the sample and the time that the first sample was taken during the secondary progressive phase (timepoint 0). To assess whether the relationship between the protein and the time from conversion is different before and after conversion, an interaction term—Time from conversion ∗ status—was included in the linear regression model (status = relapsing–remitting multiple sclerosis or secondary progressive multiple sclerosis).

To get insights into the cell types that might contribute to the differences in the extracellular miRNA profiles between different subgroups, we calculated cell type–enrichment scores from miRNA data.^21^ This approach uses the observation that miRNA profiles are highly variable between cell types.^22,23^ The top 10 miRNAs expressed in immune and neurological cell types were extracted from the miRNA atlas described by De Rie *et al*.^22^ These ‘cell type signature’ miRNAs were then used as input to perform single-sample enrichment analyses to calculate the miRNA-based cell type enrichment score [R-package GSVA (version 1.34.0, https://github.com/rcastelo/GSVA)].

## Results

### Descriptive characteristics

The descriptive characteristics of cohort I (cross-sectional case–control cohort, *n* = 105) and cohort II (prospective multiple sclerosis cohort, *n* = 93) are displayed in Supplementary Table 1. The baseline characteristics of the patients in cohort I did not differ significantly between multiple sclerosis and controls. As expected, patients with progressive forms of multiple sclerosis were, on average, older than those with relapsing–remitting multiple sclerosis. None of the patients contributing to cohort I were treated with disease-modifying drugs.

The male:female ratio, age at the first sample, age at diagnosis and duration of follow-up of the subjects in cohort II did not differ between participants who converted to secondary progressive disease (converters) and those who stayed in the relapsing–remitting phase (*P*-values = 1, 0.9, 0.7 and 0.6, respectively). For the non-progressing relapsing–remitting group, we selected patients based on the highest age at inclusion to minimize age differences compared to progressive multiple sclerosis patients. Consistent with cohort I, the average age of patients with primary progressive disease was higher than the other subgroups. Information about disease-modifying medication in cohort II is displayed in Supplementary Table 1. In this cohort, with the low percentage of therapy usage, we did not have the power to look for associations between disease-modifying drugs and biomarker expression nor between disease-modifying drug use and disease progression. As almost all converters were on stable medication during the conversion from relapsing–remitting to secondary progressive multiple sclerosis, a bias caused by medication switches around conversion is deemed unlikely.

### Protein profiles of CSF and serum samples differ

In cohort I, paired CSF and serum samples were available for the same individuals. This allowed us to check whether the protein levels detected in serum and CSF were correlated. Most proteins (72/84 proteins) displayed significantly higher levels in serum than in CSF from the same individual (see Supplementary Table 2), with the exception of seven [IL8, adhesion G-protein coupled receptor G1 (ADGRG1), carbonic anhydrase 9 (CAIX), decorin (DCN), tumour necrosis factor receptor superfamily member 21 (TNFRSF21), CD83 and pleiotrophin (PTN)]. For most proteins, the correlation in levels between serum and CSF was low (Supplementary Table 2 and Supplementary Fig. 2A). Only MHC class I polypeptide-related sequences A/B (the assay detects both MICA and MICB) showed a high correlation between serum and CSF (Supplementary Fig. 2B). Protein profiles within samples of the same type (within-sample correlation) were more highly correlated than protein profiles within the same individual (CSF versus serum) (Supplementary Fig. 2C and D). Altogether, these analyses demonstrated that the protein profiles of serum and CSF samples differ. The weak correlations between the protein levels in CSF and serum from the same individual suggest that serum and CSF are separate compartments in which biomarkers of different disease-related processes might be detected.

### CSF-specific multiple sclerosis biomarkers

We identified 13 multiple sclerosis-specific proteins in CSF (multiple sclerosis patients versus controls, FDR < 0.1) (Supplementary Fig. 3, Supplementary Table 3A). After correcting for age and sex, four proteins passed the FDR < 0.1 threshold: CD27, C-X-C motif chemokine 13 (CXCL13), granzyme (GZM) A and IL7. We did not observe any proteins to be significantly different between relapsing–remitting multiple sclerosis and secondary progressive multiple sclerosis, but most of the multiple sclerosis-associated markers (11/13) showed a trend towards a higher expression in relapsing–remitting multiple sclerosis patients.

The predictive value of biomarkers identified in a discovery cohort should ideally be validated in a second independent cohort; however, we did not have CSF samples for the patients from cohort II. Nonetheless, our discovery results confirmed a number of previously described multiple sclerosis-specific markers [multiple sclerosis patients versus controls; CXCL13, CD27, IL12, CD5, C-C motif chemokine 3 (CCL3) (also known as macrophage inflammatory protein 1-alpha (MIP1-alpha)), CXCL11, CXCL10, IL7 and tumour necrosis factor ligand superfamily member 14 (TNFSF14)].^24-26^ These nine proteins are consistent with previous studies.

**Figure 2:**
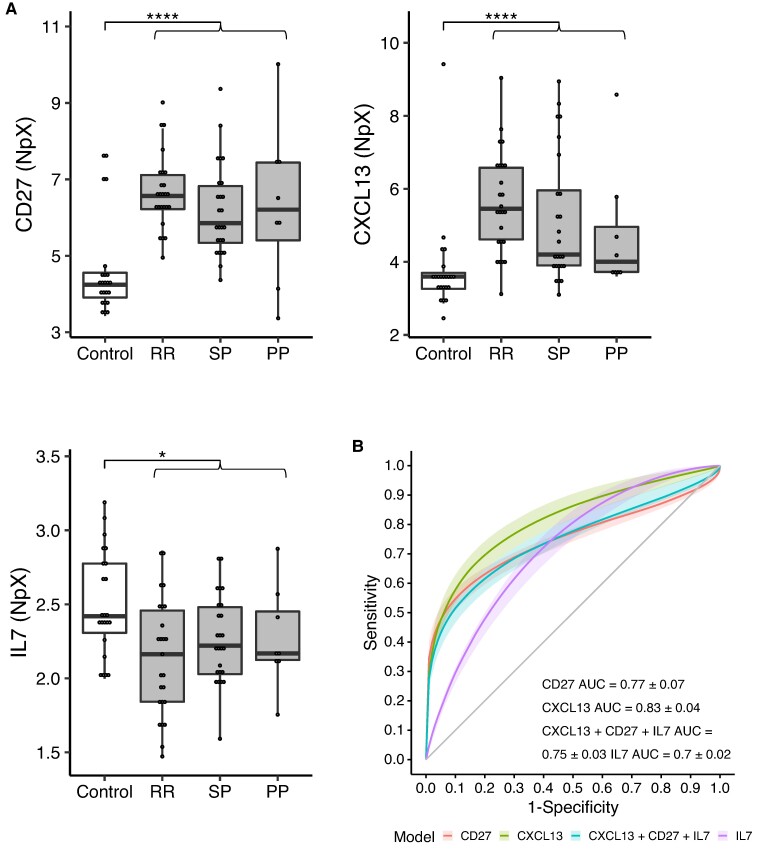
**Protein levels differ between multiple sclerosis and controls in cerebrospinal fluid.** (**A**) 3/13 proteins are significantly different between multiple sclerosis (grey) and controls (white) in CSF. RR: relapsing–remitting multiple sclerosis; SP: secondary progressive multiple sclerosis; PP: primary progressive multiple sclerosis. Test used: *t*-test (CD27 and IL7), ANOVA (CXCL13) (see also Supplementary Table 3A). ****FDR < 0.0001, *FDR < 0.05. Protein levels are shown as an NpX (normalized protein expression) unit. In the dotplot, each dot represents the protein level of an individual. (**B**) 10× cross-validated receiver operating characteristic curve for the three markers (CD27, CXCL13 and IL17) that were selected after the backward feature selection to be the best predictors of multiple sclerosis versus controls in CSF (sample size: *n* = 58 multiple sclerosis versus controls *n* = 23) and the receiver operating characteristic curve of the model combining these three biomarkers.

We then assessed which of the 13 multiple sclerosis-specific biomarker candidates had the highest predictive value in our cohort by testing the predictive power of both individual biomarkers and biomarker combinations. Here, we found that CD27, CXCL13 and IL7 had the highest predictive power, but combining these biomarkers did not lead to further increases in performance (Fig. 2A). Of the three biomarkers, CXCL13 reached the highest AUC (0.83 ± 0.04) (Fig. 2B).

### Proteins in serum as biomarkers for multiple sclerosis and multiple sclerosis subtypes

Because drawing blood is less invasive than obtaining CSF by lumbar puncture, we also compared the protein panel in the serum samples between MS patients and controls (cohort I) and between the MS subtypes (cohorts I and II).

In our case–control cohort, one serum protein, angiopoietin-1 receptor (TIE2), showed significantly higher levels in multiple sclerosis patients compared to controls (FDR = 0.04) and remained significant after correction for sex and age (FDR = 0.01) (Supplementary Table 3B). Two other proteins, ADGRG1 and PTN, were significant across the groups (controls and multiple sclerosis subtypes) according to Kruskal–Wallis/ANOVA test (FDR = 0.008 and FDR = 0.046, respectively). In the post-hoc analyses, TIE2, PTN and ADGRG1 all had significantly lower levels in relapsing–remitting patients compared to control individuals (FDR = 0.005, FDR = 0.034 and FDR = 0.034, respectively). PTN and ADGRG1 were also significantly different between relapsing–remitting multiple sclerosis and secondary progressive multiple sclerosis (FDR = 0.0053 and FDR = 0.041, respectively). For the three serum markers, the largest differences were observed between relapsing–remitting multiple sclerosis and controls.

**Figure 3:**
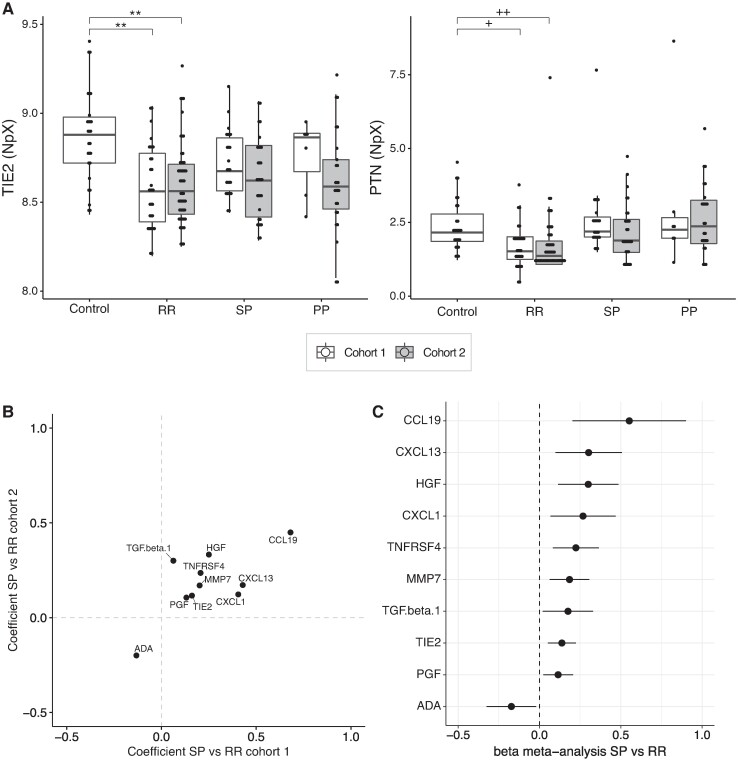
**In serum, several proteins differ between controls and relapsing–remitting (RR-MS) and/or between relapsing–remitting and secondary progressive multiple sclerosis.** RR: relapsing–remitting multiple sclerosis; SP: secondary progressive multiple sclerosis; PP: primary progressive multiple sclerosis. (**A**) Protein levels of TIE2 and PTN in controls differ from RR-MS of cohorts I and II corrected for age and sex, although PTN does not pass the significance level after multiple-testing correction. Test used: *t*-test (TIE2), ANOVA (PTN) (see also Supplementary Table 3B). ****FDR < 0.0001, ***FDR < 0.001, **FDR < 0.01, *FDR < 0.05. ^+^*P* = 0.03, FDR = 0.16. ^++^*P* = 0.01, FDR = 0.14. Protein levels are shown as an NpX (normalized protein expression) unit. In the dotplot, each dot represents the protein level of an individual. (**B**, **C**) Ten proteins are significantly different between RR-MS and SP-MS in a meta-analysis combining the analyses in both cohorts independently. (**B**) Coefficients of the comparison of RR-MS and SP-MS in cohorts I and II for these 10 proteins. Coefficients were corrected for age and sex. A positive coefficient indicates higher levels in SP-MS compared to RR-MS. (**C**) Results of the meta-analysis combining the two analyses summarized in **B**, displayed as the beta of the meta-analysis and 95% confidence interval.

We next assessed whether the trends observed in the serum samples in relapsing–remitting multiple sclerosis versus controls in the exploratory cohort could also be observed in cohort II. We performed the analyses for all measured proteins in cohort II. Because no controls were available in cohort II (multiple sclerosis cohort), the controls of cohort I were compared with the relapsing–remitting multiple sclerosis patients of cohort II (see Methods and Supplementary Fig. 1). TIE2 and PTN were the only proteins that showed a nominal *P*-value < 0.05 between controls and relapsing–remitting multiple sclerosis in cohorts I and II after correction for sex and age (Supplementary Table 4A). Of these, only TIE2 had an FDR < 0.1 after multiple-testing correction in both cohorts (cohort I TIE2: FDR = 1.4 ∗ 10^−3^, cohort II: TIE2 FDR = 2.5 ∗ 10^−04^). Figure 3A shows the trends of TIE2 and PTN in both cohorts.

To assess whether we could identify biomarkers that distinguish between relapsing–remitting multiple sclerosis and secondary progressive multiple sclerosis, we compared differences within the two cohorts independently while correcting for sex and age (Supplementary Table 4B). All proteins that showed a trend (nominal *P*-value < 0.1) were then combined in a meta-analysis (Supplementary Table 4B). Of the 30 proteins tested, 10 displayed significance in the meta-analysis: TIE2, adenosine deaminase, placenta growth factor, transforming growth factor beta-1 proprotein, matrilysin, TNFRSF4, CXCL1, hepatocyte growth factor (HGF), CXCL13 and CCL19. Figure 3B shows the concordance between the coefficients (estimates) of these 10 proteins between relapsing–remitting multiple sclerosis and secondary progressive multiple sclerosis in cohorts I and II (Fig. 3C). These 10 proteins in serum that differ between relapsing–remitting multiple sclerosis and secondary progressive multiple sclerosis are the top candidate biomarkers.

### High levels of cytotoxic and regulatory T-cell molecule are associated with primary progressive multiple sclerosis

Primary progressive multiple sclerosis is characterized by a progressive course from disease onset. We assessed whether the biomarkers described above could also be identified in primary progressive multiple sclerosis. As only a limited number of patients with primary progressive multiple sclerosis were included in cohort I, the analyses with primary progressive multiple sclerosis were only performed in cohort II. Serum levels of cytotoxic and regulatory T-cell molecule were significantly different in primary progressive multiple sclerosis when compared to relapsing–remitting multiple sclerosis (nominal *P*-value = 0.001, FDR = 0.095, corrected for sex and age). No significant differences were observed between primary and secondary progressive multiple sclerosis. We next assessed the similarities in the biomarker profile between primary progressive multiple sclerosis and the other subtypes of multiple sclerosis, especially for the 10 markers that were significant in the meta-analysis described above (relapsing–remitting versus secondary progressive multiple sclerosis). Here, we observed that the difference between relapsing–remitting multiple sclerosis and secondary progressive multiple sclerosis was more pronounced than the differences between relapsing–remitting and primary progressive multiple sclerosis (Supplementary Fig. 4). Based on these inflammatory protein markers, primary progressive multiple sclerosis lies on the spectrum between relapsing–remitting and secondary progressive multiple sclerosis.

### Granzymes in serum as biomarkers for conversion from relapsing–remitting multiple sclerosis to secondary progressive multiple sclerosis

**Figure 4:**
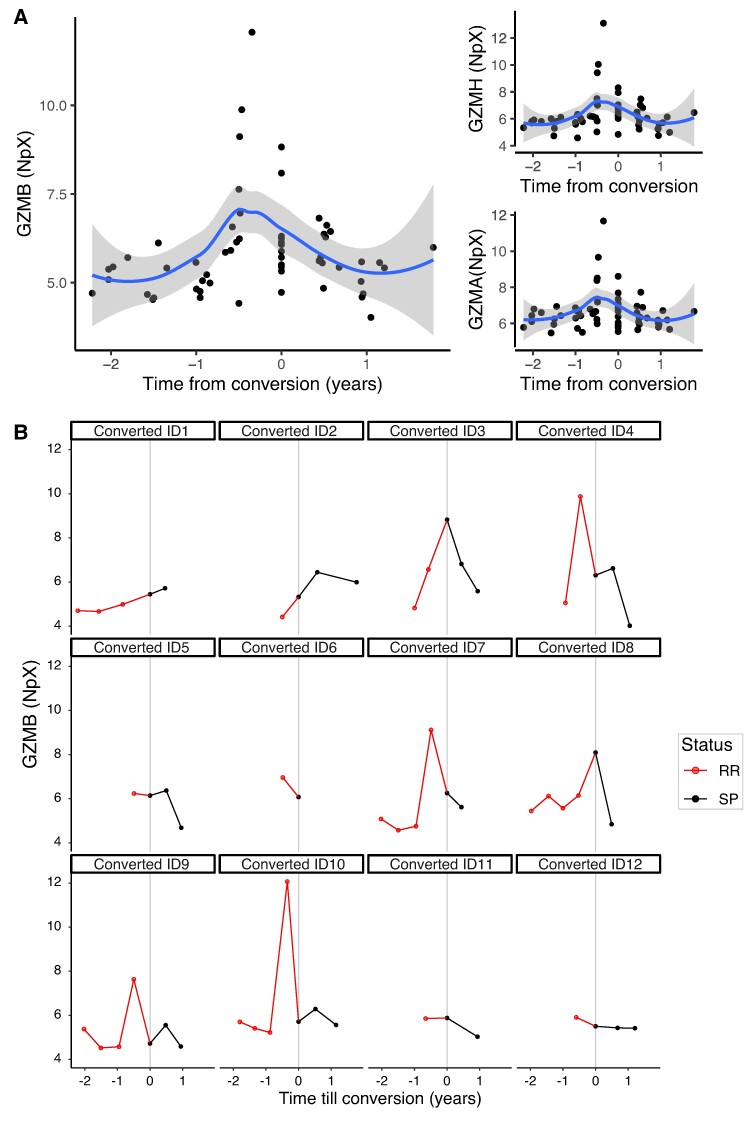
**GZMB peaks around conversion from RR-MS to SP-MS.** RR: relapsing–remitting multiple sclerosis; SP: secondary progressive multiple sclerosis; protein levels are shown as an NpX (normalized protein expression) unit. (**A**) Levels of granzyme (GZM) B/A/H in all samples of the individuals who converted from RR-MS to SP-MS. The blue line shows a smooth local regression and a 95% confidence interval (grey). Time until conversion (in years) was defined based on the first sample classified as secondary progressive disease. (**B**) Trends for GZMB per individual for the individuals that converted from RR-MS to SP-MS during the study.

The longitudinally collected samples of the converters (see Fig. 1) allowed us to look for markers for the conversion from relapsing–remitting to secondary progressive multiple sclerosis. In total, 6/82 proteins tested were significantly associated (FDR < 0.1) with the ‘time from conversion’. Three of these showed increasing levels (GZMB, GZMH and GZMA), and three showed decreasing levels [pro-epidermal growth factor (EGF), CCL17 and CD40 ligand (CD40L)] towards conversion. We identified a significant interaction term for GZMB and GZMH (FDR = 0.078 and FDR = 0.0973, respectively), which indicates that the levels of these two proteins increased up until conversion, and then decreased after the patients converted to secondary progressive multiple sclerosis (GZMB is shown in Fig. 4A). Looking at the trends per individual (Fig. 4B), the GZMB levels peak around the point of conversion in at least 6/12 individuals.

In conclusion, six protein candidates (GZMB, GZMH, GZMA, EGF, CCL17 and CD40L) are associated with the time from conversion. To validate that this is a specific change related to the conversion, we also assessed the trend for these markers in the relapsing–remitting multiple sclerosis patients who did not convert. In this analysis, we did not observe significant changes for GZMH (nominal *P* = 0.68), GZMA (nominal *P* = 0.19) and GZMB (nominal *P* = 0.17) in the relapsing–remitting multiple sclerosis patients, implying that these markers are indeed related to the conversion. In contrast, the levels of EGF (nominal *P* = 0.005), CCL17 (nominal *P* = 0.03) and CD40.L (nominal *P* = 0.03) did change in relapsing–remitting multiple sclerosis as well, implying that these proteins are not suitable as markers for conversion.

### miRNA profiles differ between CSF samples and serum

**Figure 5:**
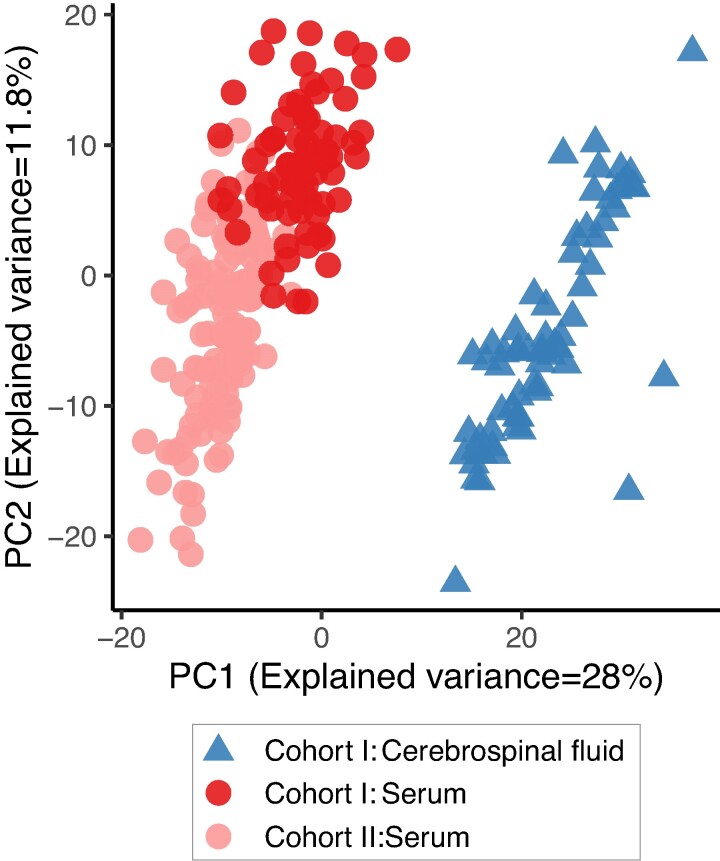
Principal component analysis of miRNA profiles shows that the CSF samples cluster away from the serum samples.

Because circulating miRNAs have emerged as promising biomarkers, we also performed miRNA sequencing on both cohorts and found large differences when comparing the miRNA profiles of the CSF samples to those in the serum samples (Fig. 5 and Supplementary Fig. 5A). Principal component analysis (Fig. 5) demonstrates that most of the variation in the data is explained by the sample type in line with previous observations.^27,28^ The diversity and total number of miRNAs detected (library size) were significantly lower in CSF than in serum (Supplementary Table 1). Based on previous reports, we had expected CSF samples to yield miRNA libraries different from serum. Indeed, the nervous system-specific miRNAs, miR-1298, miR-9 and miR-1911, could be detected in CSF samples (mean raw read counts in CSF 225, 77 and 52, respectively), but not in serum (fewer than two raw counts). Another brain-specific miRNA, miR-204-5p, was the most abundant miRNA in CSF, but had far lower levels in serum (ranked 173rd). Overall, the miRNA profile in CSF displayed ‘compartment-specificity’ and was enriched for miRNAs expressed in the brain.

### Multiple sclerosis stage-specific miRNAs in CSF

In CSF, we did not identify miRNAs that were significantly differently expressed between multiple sclerosis and controls. However, seven miRNAs (miR-451a, miR-16-2-3p, miR-9-5p, miR-15a-5p, miR-144-3p, miR-100-5p and miR-210-3p) were significantly higher in secondary progressive multiple sclerosis compared to relapsing–remitting multiple sclerosis (Supplementary Table 5). Thus, in contrast to the protein profile in CSF, the miRNA profile does not show large differences between multiple sclerosis and controls, but shows differences between multiple sclerosis subtypes.

### Multiple sclerosis and multiple sclerosis stage-specific miRNAs in serum

**Figure 6:**
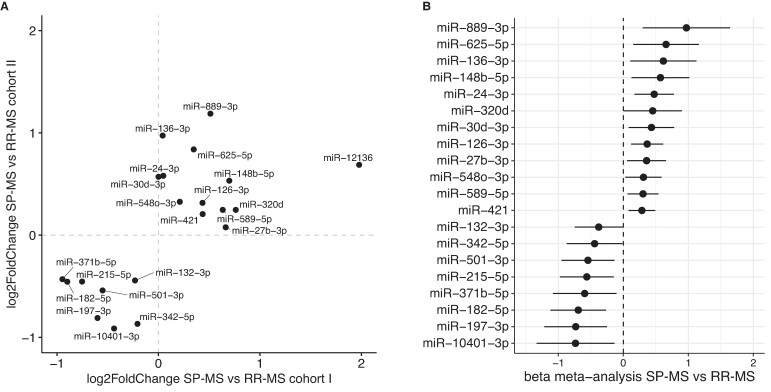
**Meta-analysis reveals the miRNA biomarker candidates between relapsing–remitting (RR-MS) and secondary progressive multiple sclerosis (SP-MS).** A total of 16 miRNAs were significantly different between RR-MS and SP-MS in the meta-analysis combining the analyses performed in both cohorts independently. Additionally, miR-12136 was significantly different in both cohorts (FDR < 0.1) and significant using a random-effect analysis (nominal *P*-value = 0.05). (**A**) log2FoldChanges of the comparison of RR-MS and SP-MS in cohorts I and II for these 17 miRNAs. The coefficients were corrected for age, sex and technical batch. A positive coefficient indicates higher levels in SP-MS compared to RR-MS. (**B**) Results for the 16 miRNAs significant in the meta-analysis (shown as beta of the meta-analysis, 95% confidence interval).

In sera from cohort I, we observed significant differences between multiple sclerosis and controls. Specifically, of the 51 differing miRNAs, 29 were downregulated, and 22 were upregulated in multiple sclerosis (Supplementary Table 6). We then compared miRNA levels in relapsing–remitting multiple sclerosis and secondary progressive multiple sclerosis samples in both cohorts independently (Supplementary Table 7). The differentially measured miRNAs (relapsing–remitting multiple sclerosis versus secondary progressive multiple sclerosis) were then combined in a meta-analysis (Supplementary Table 7). In total, 16 miRNAs were significantly differentially detected in the meta-analysis (Fig. 6), making them the top candidate biomarkers to detect differences between relapsing–remitting multiple sclerosis and secondary progressive multiple sclerosis in serum.

As only limited numbers of patients with primary progressive multiple sclerosis were included in cohort I, we could only perform the analyses with primary progressive multiple sclerosis in cohort II. No miRNAs were significantly different when comparing primary progressive multiple sclerosis to relapsing–remitting, and only one miRNA (miR-1908-3p) was significantly different between primary and secondary progressive multiple sclerosis (Supplementary Table 8A). Data in Supplementary Fig. 6 illustrate that, for miRNA markers, primary progressive multiple sclerosis lies on the spectrum between relapsing–remitting and secondary progressive multiple sclerosis.

In conclusion, we identified 51 multiple sclerosis-specific miRNAs in serum, including 16 candidate biomarkers for discriminating relapsing–remitting from secondary progressive multiple sclerosis.

### Longitudinal analyses reveal miRNAs associated with time from conversion

**Figure 7:**
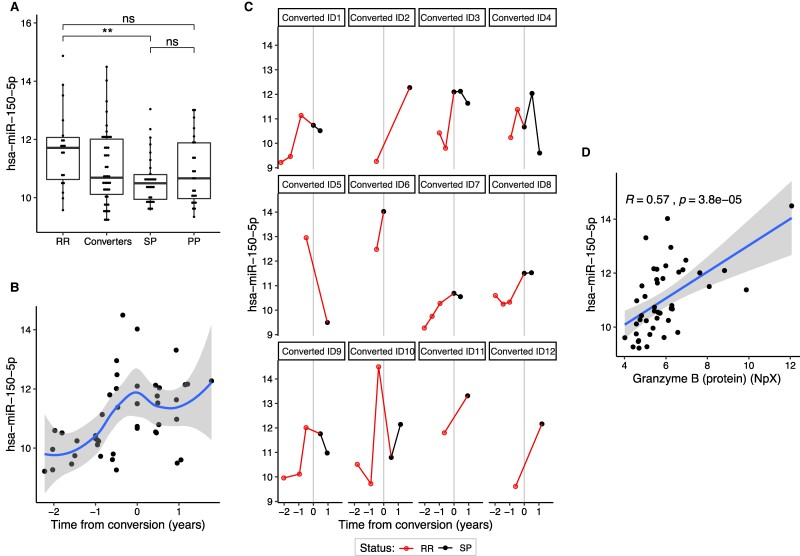
**Level of miR-150 in serum is associated with time from conversion and positively correlated to granzyme B (GZMB) protein levels in serum.** RR: relapsing–remitting multiple sclerosis; SP: secondary progressive multiple sclerosis; PP: primary progressive multiple sclerosis. (**A**) miRNA-150 is significantly lower in the secondary progressive group compared to the relapsing–remitting patients in the differential expression analysis (**FDR = 0.001). For visualization purposes, the miRNA levels were a normalized regularized log function. In the dotplot, each dot represents the miRNA level of an individual. (**B**) Within the patients that converted from RR to SP during the study, miR-150 is associated with time from conversion (FDR = 0.0004, see Supplementary Table 8B). The blue line shows a smooth local regression and 95% confidence interval (grey). Time from conversion (in years) was defined based on the first sample that was classified as secondary progressive disease. (**C**) Trends in miRNA-150 per individual for the individuals who converted from RR-MS to SP-MS during the study. (**D**) miRNA-150 and GZMB protein levels are correlated (Pearson correlation) in the serum samples of individuals who converted during the study. Protein levels are shown as an NpX (normalized protein expression) unit.

To find miRNAs that were associated with conversion, we analysed the miRNA profiles of the samples from the converters. This identified 100 miRNAs that were associated (FDR < 0.1) with ‘time from conversion’ (Supplementary Table 8B). For 24 of the 100 miRNAs, the trend before and after conversion was significantly different, as indicated by a significant interaction term. As this was a very high number of significant miRNAs associated with the time from conversion, we sought to narrow down these candidates to eliminate false-positive hits. To do so, we checked for correlations between these miRNAs and GZMB, the protein that showed the clearest peak around conversion. Of the 24 miRNAs with a different trend before and after conversion, 10 also showed a significant correlation with GZMB. These miRNAs included the immune-related miRNA-150 that peaks around the conversion from relapsing–remitting to secondary progressive multiple sclerosis (Fig. 7). These 10 miRNAs were not among the 16 biomarker candidates that discriminated between relapsing–remitting and secondary progressive multiple sclerosis, indicating that these 10 miRNAs were markers whose levels peak around conversion, and then normalize afterwards.

### miRNAs associated with conversion to secondary progressive multiple sclerosis are immune cell-related

The observation that GZMB peaks in serum around conversion led us to hypothesize that the conversion of relapsing–remitting to secondary progressive multiple sclerosis coincides with an immune cell-driven event. Alongside the peak GZMB, we observed miRNA profile shifts related with the conversion. We investigated whether these extracellular miRNA shifts around conversion also reflect immune cell-driven events. Because miRNA expression profiles are highly cell type-specific, we can make inferences about the potential source cell types of the extracellular miRNAs^22,23^

First, we assessed whether the miRNAs associated with GZMB levels in converters (see Supplementary Table 8B) are known to be enriched in immune cells or nervous system-related cells using a previously published repository of the miRNA expression data in purified human cell types.^22^ We observed that many of the miRNAs that were significantly positively associated with GZMB are known to be enriched in immune cells (Supplementary Fig. 7). In contrast, miRNAs that showed a negative association with GZMB showed a trend towards being more enriched in nervous system-related cells (Supplementary Fig. 7). This supported our hypothesis that the miRNA profile shifts due to immune cell-related events around conversion.

To further explore this hypothesis, we used another approach that looks for cell type-specific miRNA signatures in miRNA libraries. This method calculates the enrichment score of miRNAs related to a specific cell type in that particular sample. As a proof-of-concept, we first calculated these miRNA-based enrichment scores in cohort I for CSF and serum samples. As expected, the miRNA libraries for CSF showed higher enrichment scores for miRNAs from brain-related cell types (Supplementary Fig. 8), whereas the miRNA libraries for serum were more enriched for miRNAs from peripheral blood mononuclear cells.

**Figure 8:**
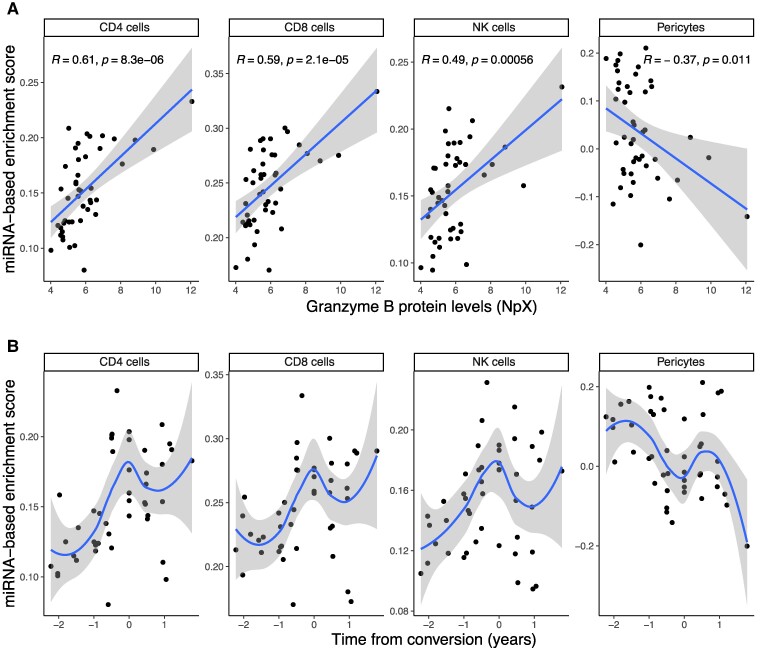
**miRNA-based enrichment scores for several cell types in serum are associated with (A) granzyme B (GZMB) protein levels [blue line shows the linear regression, ±95% confidence interval (grey)] and (B) time from conversion [blue line shows a smooth local regression, 95% confidence interval (grey)].** Plots show the samples from individuals who converted from relapsing–remitting to secondary progressive multiple sclerosis during the study. Protein levels are shown as an NpX (normalized protein expression) unit.

Next, we calculated the miRNA-based enrichment scores in the converters and associated these to GZMB and the time from conversion. The GZMB levels were significantly positively associated with the miRNA-based enrichment scores for CD4+ cells, CD8+ cells and NK cells (Fig. 8A). The miRNA-based enrichment scores for pericytes, a cell type wrapping microvasculature in the brain that is important in the maintenance of the blood–brain barrier, were negatively associated with GZMB (Fig. 8A). The miRNA-based enrichment scores for these cell types were also significantly associated with time from conversion (CD4+ FDR = 0.02, CD8+ FDR = 0.02, NK cells FDR = 0.02, pericytes FDR = 0.03) (Fig. 8B). Thus, the miRNA profiles around conversion were enriched for miRNAs associated with certain immune cells and depleted for miRNAs associated with pericytes. These data further support that conversion from relapsing–remitting to progressive multiple sclerosis is an immune cell-driven event, and not simply an emerging predominance of neurodegeneration over inflammation.

## Discussion

Because treatment modalities differ, there is a need for non-invasive biomarkers that can help discriminate the relapsing–remitting subtype of multiple sclerosis from the progressive forms of multiple sclerosis. In the current study, we performed an extensive analysis of proteins and miRNAs in serum and paired CSF samples from multiple sclerosis patients and controls and in a set of prospectively collected longitudinal serum samples of a multiple sclerosis cohort.

We observed that miRNA and protein profiles were highly dependent on their compartment of origin (serum versus CSF), and we were able to validate several proteins previously implicated as multiple sclerosis biomarkers in CSF.^24-26^ We also uncovered novel biomarker candidates in blood that differentiate between relapsing–remitting and secondary progressive multiple sclerosis. Interestingly, we identified a clear inflammatory signature in blood that peaks around the conversion from the relapsing–remitting phase to the secondary progressive phase of disease, implying that the immune response is involved in the progression from the ‘inflammatory state’ of multiple sclerosis (relapsing–remitting multiple sclerosis) to the ‘neurodegenerative state’ of multiple sclerosis (secondary progressive multiple sclerosis). To our knowledge, this is one of the first studies to use both cross-sectional and longitudinal sampling that was designed specifically for an in-depth analysis of both miRNA and protein biomarkers around the multiple sclerosis conversion timepoint.

One of the strengths of our explorative study is the availability of paired serum and CSF samples. Our results reconfirmed that both protein and miRNA profiles in CSF are not simply a reflection of serum profiles, and vice versa. Most proteins showed a low correlation when comparing compartments, with the exception of MHC class I-like proteins (MIC) A/B, which correlated strongly between serum and CSF. The *MICB* gene expression had been previously associated with multiple sclerosis.^29^ The very strong correlation between compartments could suggest there is a free exchange of this protein, despite the presence of the blood–brain barrier. Based on previous reports, we expected the CSF samples to yield different miRNA libraries compared to serum. We confirmed the presence of the nervous tissue and CSF-specific miRNAs miR-1298, miR-9, miR-1911 and miR-204-5p, which validated the quality and ‘compartment-specificity’ of our miRNA libraries.^27,28,30-33^ Together, our miRNA and protein profiles confirmed that CSF and blood are two clearly separate compartments, most likely reflecting a functioning blood–brain barrier. However, despite the blood–brain barrier, we still picked up biomarkers specific for multiple sclerosis (and multiple sclerosis subtypes) in the serum samples. This is important in the search for novel biomarkers because collecting blood is less invasive and easier than collecting CSF, especially when planning longitudinal sampling.

miRNAs are a relatively new class of biomarker candidates. In our serum samples, we identified 51 multiple sclerosis-specific miRNAs and 16 miRNAs that discriminated relapsing–remitting from secondary progressive multiple sclerosis. In CSF, we identified seven miRNAs that showed higher levels in secondary progressive multiple sclerosis compared to relapsing–remitting multiple sclerosis. To date, the results of various miRNA studies and the association of miRNAs with specific diseases and disease stages have been difficult to replicate probably due to the large methodological and analytical differences between studies.^34^ The few miRNAs that have been most consistently linked to multiple sclerosis include miR-223 and miR-23. Both these miRNAs display decreased levels in our multiple sclerosis blood samples.^13,34-36^ Still, our findings need to be replicated in independent studies using uniform methodology before the biomarkers that we suggest in our study can be translated into biomarkers that can be used clinically.

Our results validated multiple multiple sclerosis-specific protein biomarkers in CSF (e.g. CD27 and CXCL13).^24-26^ In our serum samples, the levels of these proteins could not differentiate multiple sclerosis samples from controls nor were they biomarkers for multiple sclerosis subgroups in line with previous findings.^24^ Neurofilament light chain (NFL) belongs to a class of proteins released when there is neuro-axonal damage and can be detected in both CSF and blood as a biomarker for multiple sclerosis.^37-39^ In the current study, we did not measure NFL.

In contrast to CSF, with NFL as an exception, very few protein biomarkers for multiple sclerosis have been reported in blood. Huang *et al*.^24^ proposed two markers for multiple sclerosis: HGF and oncostatin M. We measured HGF, but did not confirm the earlier findings. Our results uncovered 10 novel candidate biomarkers that differentiated between relapsing–remitting multiple sclerosis and secondary progressive multiple sclerosis. Because NFL cannot distinguish relapsing–remitting and secondary progressive multiple sclerosis^40^ and because of the lack of other biomarkers, these 10 candidates are of interest and need to be validated in independent studies. One of these candidates, TIE2, is of special interest from a pathophysiological point of view because it has a role in the blood–brain barrier maintenance,^41,42^ and activation of *Tie2* was shown to decrease clinical symptoms in a murine neuroinflammation model.^43^ Our study suggests that a low serum level of TIE2 is associated with relapsing–remitting multiple sclerosis, and this may be linked to a decreased blood–brain barrier function. Future studies should address how expression levels of *TIE2* in the blood–brain barrier relate to the levels of TIE2 in circulation.

For the markers that differentiated between relapsing–remitting and secondary progressive multiple sclerosis, the levels in primary progressive multiple sclerosis patients were between those of relapsing–remitting and secondary progressive multiple sclerosis. High levels of the inflammatory protein cytotoxic and regulatory T-cell molecule in blood were associated with primary progressive multiple sclerosis. These findings could support the idea that primary progressive multiple sclerosis is not on the far end of the neuroinflammation versus neuroinflammation spectrum, but instead lies on the spectrum between relapsing–remitting and primary progressive multiple sclerosis.

Longitudinal sampling and analysis are indispensable in studying a temporal process, such as conversion to secondary progressive multiple sclerosis. Using our longitudinal analysis, which involved a 6-monthly collection of serum samples, we were able to show that changes in granzyme B and immune-associated miRNA patterns mark the conversion to secondary progressive multiple sclerosis. Finding immune-associated miRNA patterns indirectly support the granzyme B protein data. Together, these findings suggest that immune-driven events are involved in the conversion from relapsing–remitting to progressive disease.

To strengthen the validity of our data and explore this hypothesis, we corrected for potential confounders. The neurologists ascertaining conversion based on clinical data were blinded to protein and miRNA results. In addition, a representative group of patients with relapsing–remitting multiple sclerosis who did not convert was used as the reference group to control the spontaneous fluctuations of biomarkers during the relapsing–remitting phase of multiple sclerosis.

Recently, GZMB-producing cells have been suggested to play a role in the disease progression in multiple sclerosis. Elevated levels of GZMB + Eomes + cytotoxic CD4+ T helper cells in blood and brain infiltrates have recently been associated with progression in multiple sclerosis.^44^ Another study reported that the *GZMB* gene expression was increased in CD8+ cells circulating in blood in SP-MS when compared to RR-MS.^45^ These findings support our theory that the GZMB peak we observed around multiple sclerosis progression could reflect disease progression in multiple sclerosis patients.

In our data, the GZMB peak was also associated with a depletion of pericyte-associated miRNAs. Pericytes play an important role in prevention of neuroinflammation by controlling the influx of immune cells into the brain through maintenance of the blood–brain barrier.^41,42^ It would be interesting to investigate whether the changes in the pericyte-associated miRNAs are indeed correlated with a change in pericyte and blood–brain barrier homeostasis. However, as we only had one cohort with longitudinal data available, these findings await final confirmation in an independently collected longitudinal cohort. Such a cohort would require the inclusion of many patients with relapsing–remitting multiple sclerosis and a sufficiently long follow-up period with frequent sampling to include a significant number of converters.

In summary, we propose novel protein and miRNA biomarker candidates for multiple sclerosis and multiple sclerosis subtypes. The GZMB peak in serum around the conversion from relapsing–remitting multiple sclerosis to secondary progressive multiple sclerosis can pave the way to new clinical applications and insights into the pathophysiology of the conversion process in multiple sclerosis. This could lead to treatments to delay or prevent conversion. Our study emphasizes the need to collect not only cross-sectional, but also longitudinal data, as only the latter approach can reveal biomarkers that allow for earlier recognition of progressive multiple sclerosis.

## Supplementary Material

fcae209_Supplementary_Data

## Data Availability

The miRNA count data and proteomics will be available as Supplementary material.
