# Supplementary material for: Potential biomarkers for multiple sclerosis stage from targeted proteomics and microRNA sequencing
Source: Brain Commun. 2024 Jun 13;6(4):fcae209. doi: 10.1093/braincomms/fcae209 (PMC11229703; doi:10.1093/braincomms/fcae209)
Supplement: fcae209_Supplementary_Data [file fcae209_supplementary_data.zip › Supplementary_Figures_and_Methods.pdf]

Supplementary Figures overview

- Supplementary Figure 1: Controlling for batch-specific effects in the proteomics data.
- Supplementary Figure 2: Comparison of protein profiles in CSF and serum.
- Supplementary Figure 3: 13 biomarker candidates differ between MS and controls in CSF.
- Supplementary Figure 4: Protein levels in primary progressive MS are between levels of relapsing-remitting and secondary progressive MS.
- Supplementary Figure 5: Comparison miRNA profiles in CSF and serum.
- Supplementary Figure 6: miRNA levels in primary progressive MS are between levels of relapsing-remitting and secondary progressive MS.
- Supplementary Figure 7: miRNA that show a positive correlation with granzyme B are enriched in immune cells.
- Supplementary Figure 8: miRNA-based enrichment scores for nervous tissue–specific cells are enriched in CSF samples versus serum samples in cohort I.

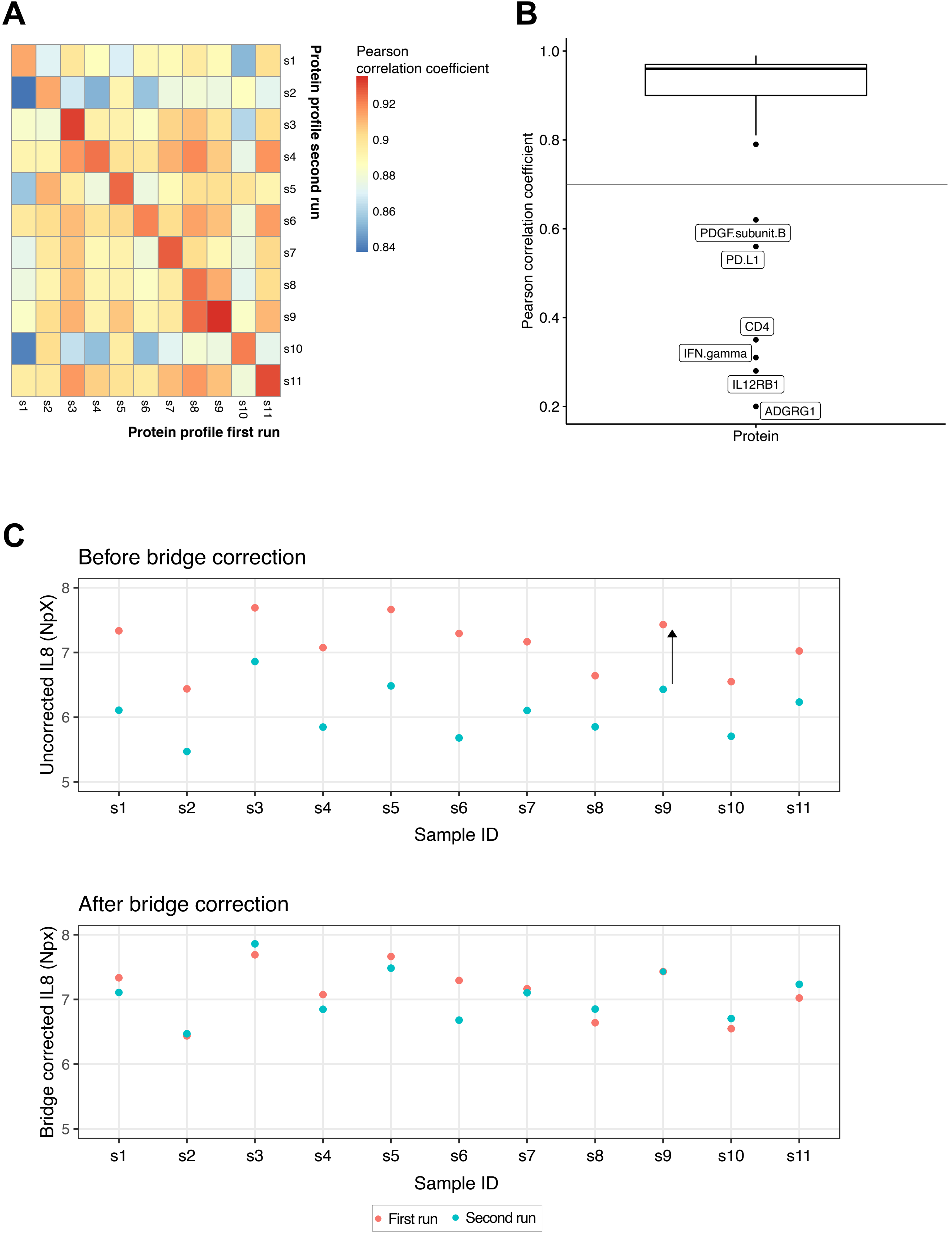

Supplementary Figure 1: **Controlling for batch-specific effects in the proteomics data.** To control for batch-specific effects, 11 random serum samples from the case-control cohort (cohort I) were profiled again (bridging samples), together with the samples from the prospective MS cohort (cohort II). **A)** The correlation between the protein profiles of the samples between the first and the second run. The correlation of the protein profile of the same sample in two different runs is high (correlation coefficient>0.9. **B)** The per protein correlation between the first and the second run (every data point represents one protein). Analytes below a correlation coefficient of 0.7 (grey line) were excluded from direct comparison of values between both cohorts (and therefore were not bridge-corrected). **C)** Shows the uncorrected (upper) and corrected (bottom) NpX values for an example protein (IL8) for the bridging samples in the first and the second run. The NpX values for IL8 of all samples analyzed in the second run were corrected for the median difference between the first and second run in the bridging samples. Back arrow in het upper panel shows this correction factor.

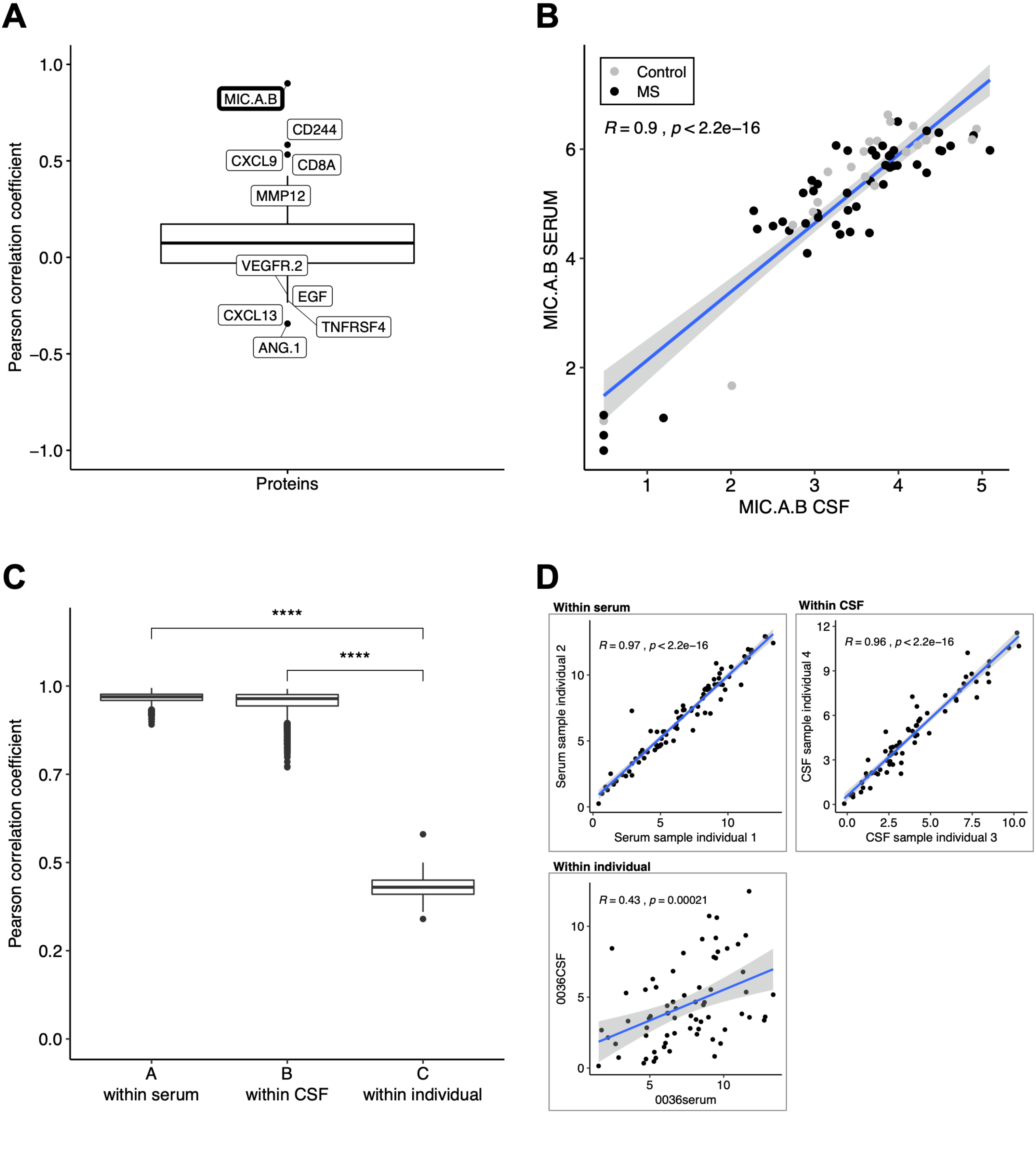

Supplementary Figure 2: comparison protein profiles in cerebrospinal fluid (CSF) and serum. A) Summarizes the correlation of protein levels between CSF and serum in the individuals for whom both sample types were available. Of the 68 proteins (detectable in both CSF and serum), only MIC.A.B showed a high correlation between serum and CSF. **B)** An example from (A) of the protein showing the highest correlation between CSF and serum. Each data point represents one individual. **C)** Correlation of the protein profiles is high across samples of the sample type (serum or CSF) (**** p <= 0.0001, Mann-Whitney U test). The correlations between the CSF and serum sample within the same individual are lower. Each data point represents the correlation coefficient within serum, CSF or within the individual, explained in: **D**) Examples of the correlations of protein profiles between individuals within the same sample type or within individuals. Of the correlations (A-C) summarized in Supplementary Figure 2C, we show one exemplary correlation. Each data point represents one protein.

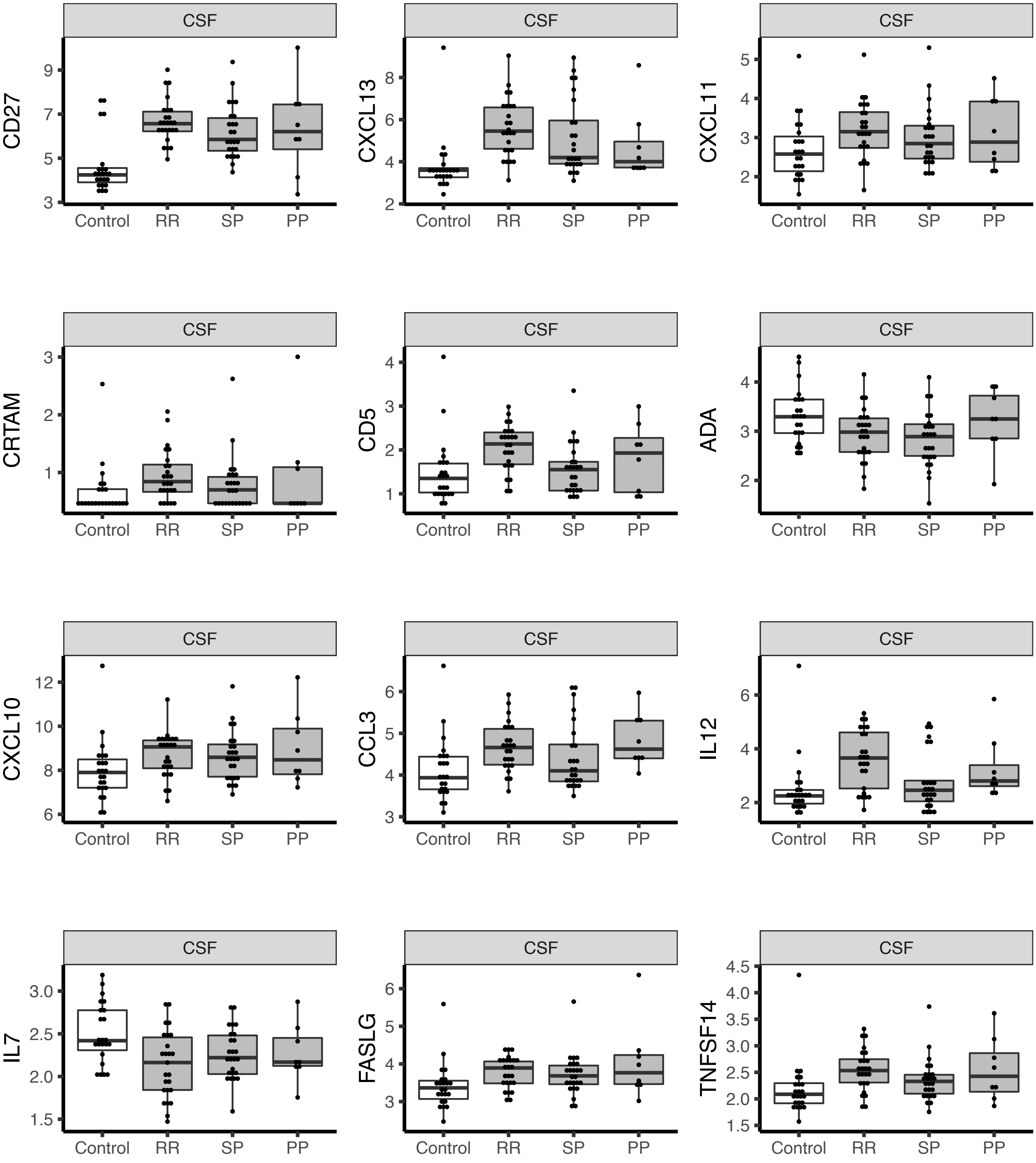

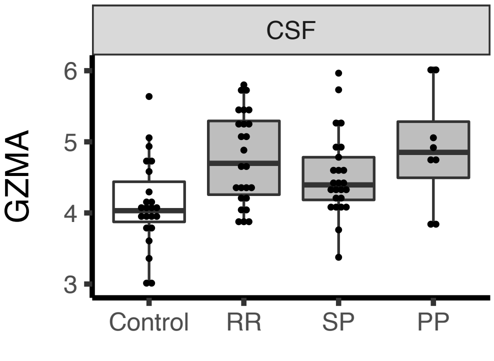

Supplementary Figure 3: 13 biomarker candidates differ between Multiple Sclerosis (MS) and controls in CSF. Protein levels are shown as NpX (Normalized Protein eXpression) unit. Note that the normalization step into NpX includes a log2 transformation. In the dotplot, each dot represents the protein level of an individual. See also Supplementary Table 3A.

Grey: MS. RR: relapsing-remitting MS. SP: secondary progressive MS. PP: primary progressive MS.

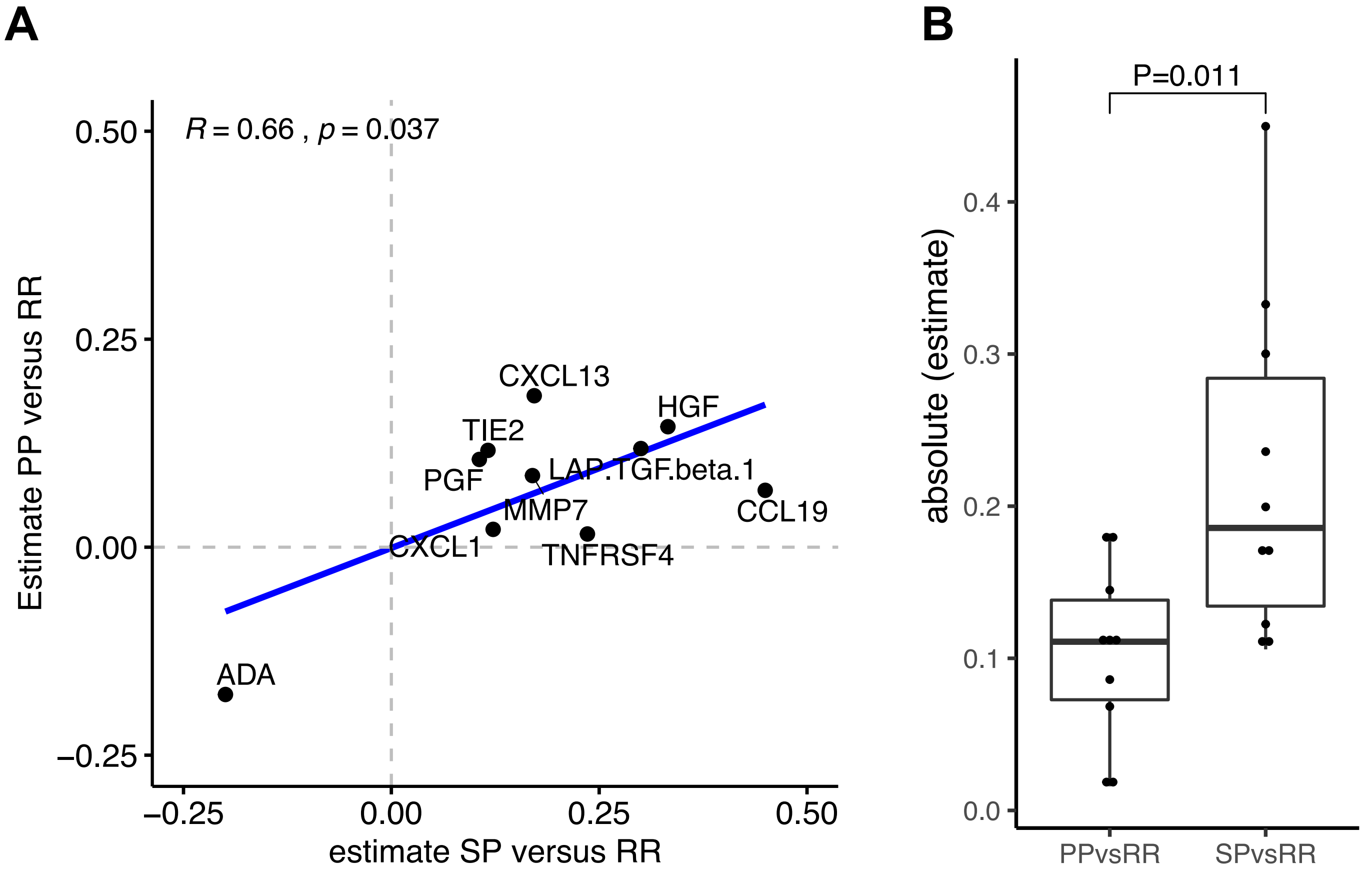

Supplementary Figure 4: Protein levels in primary progressive multiple sclerosis (MS) are between levels of relapsing-remitting and secondary progressive MS. A) Concordance between the coefficients (=estimates) between the comparisons of relapsing-remitting (RR) versus secondary progressive (SP) or primary-progressive (PP) multiple sclerosis in cohort II. The estimates are shown for the 10 proteins that were significantly different between SP and RR in the meta-analysis combining cohort I and II are shown. Analyses were corrected for age and sex. The Pearson correlation coefficient is shown in the figure. **B)** Absolute estimates for these 10 proteins are shown. The absolute estimates between SP and RR are higher than the absolute estimates between PP and RR (Mann-Whitney U test; P-value is shown in the figure).

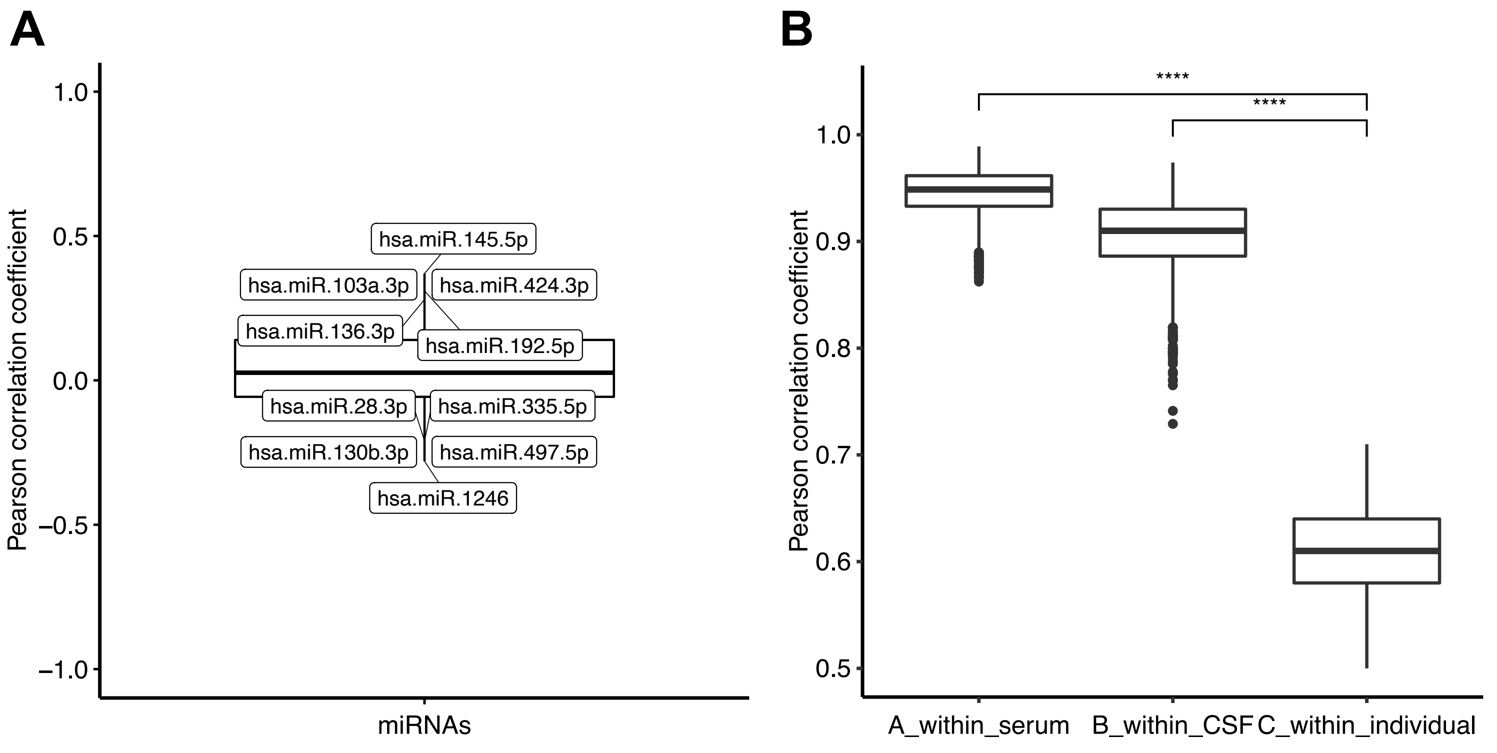

Supplementary Figure 5: Comparison miRNA profiles in CSF and serum. A) Summarizes the correlation of miRNA levels between CSF and serum in the individuals (n = 48) for whom both sample types were available. Each data point represents the pearson coefficient for the correlation between CSF and serum per miRNA. None of the 142 miRNAs that were detectable in both CSF and serum showed a high correlation (>0.5) between CSF and serum. **B)** Correlation of miRNA profiles is high across samples of the same type (serum or CSF). Correlation is lower between the CSF and serum sample from the same individual. **** P <= 0.0001, Mann-Whitney U test. Each data point represents the correlation coefficient within serum, CSF or within the individual (also explained for the proteins in Supplementary Figure 2D).

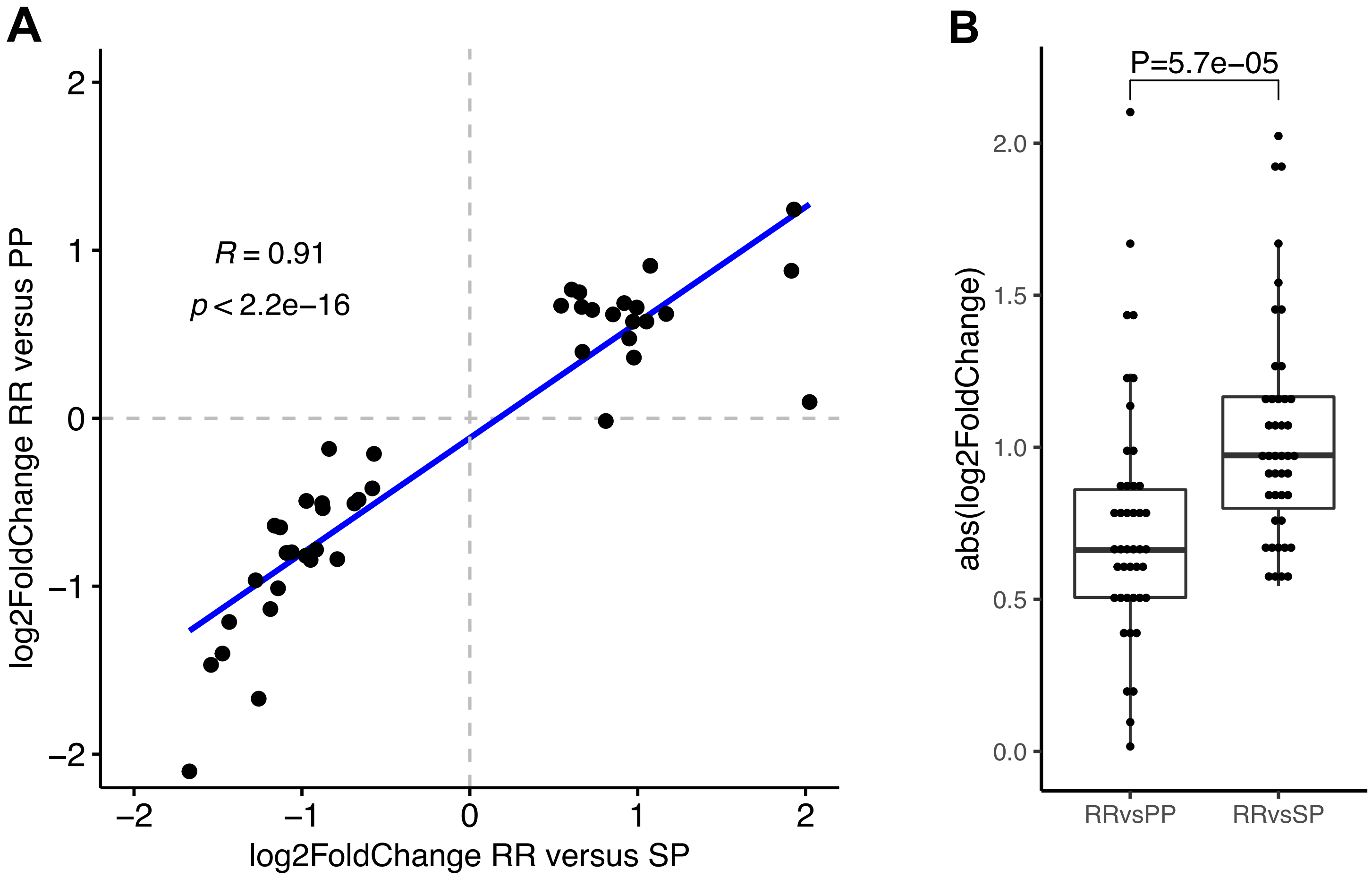

Supplementary Figure 6: miRNA levels in primary progressive multiple sclerosis (PP) are between levels of relapsing-remitting MS (RR) and secondary progressive MS (SP). A) Concodance between the log2foldchange between the comparisons of secondary progressive (SP) or primary-progressive (PP) versus relapsing-remitting (RR) in cohort II. The 43 miRNAs that were significant between SP and RR in cohort II are shown. These analyses were corrected for age, sex and technical batch. B) The absolute log2foldchanges between SP and RR are higher than the absolute estimates between PP and RR (Mann-Whitney U test). The data points indicate the 43 miRNAs that were significant between SP and RR in cohort II.

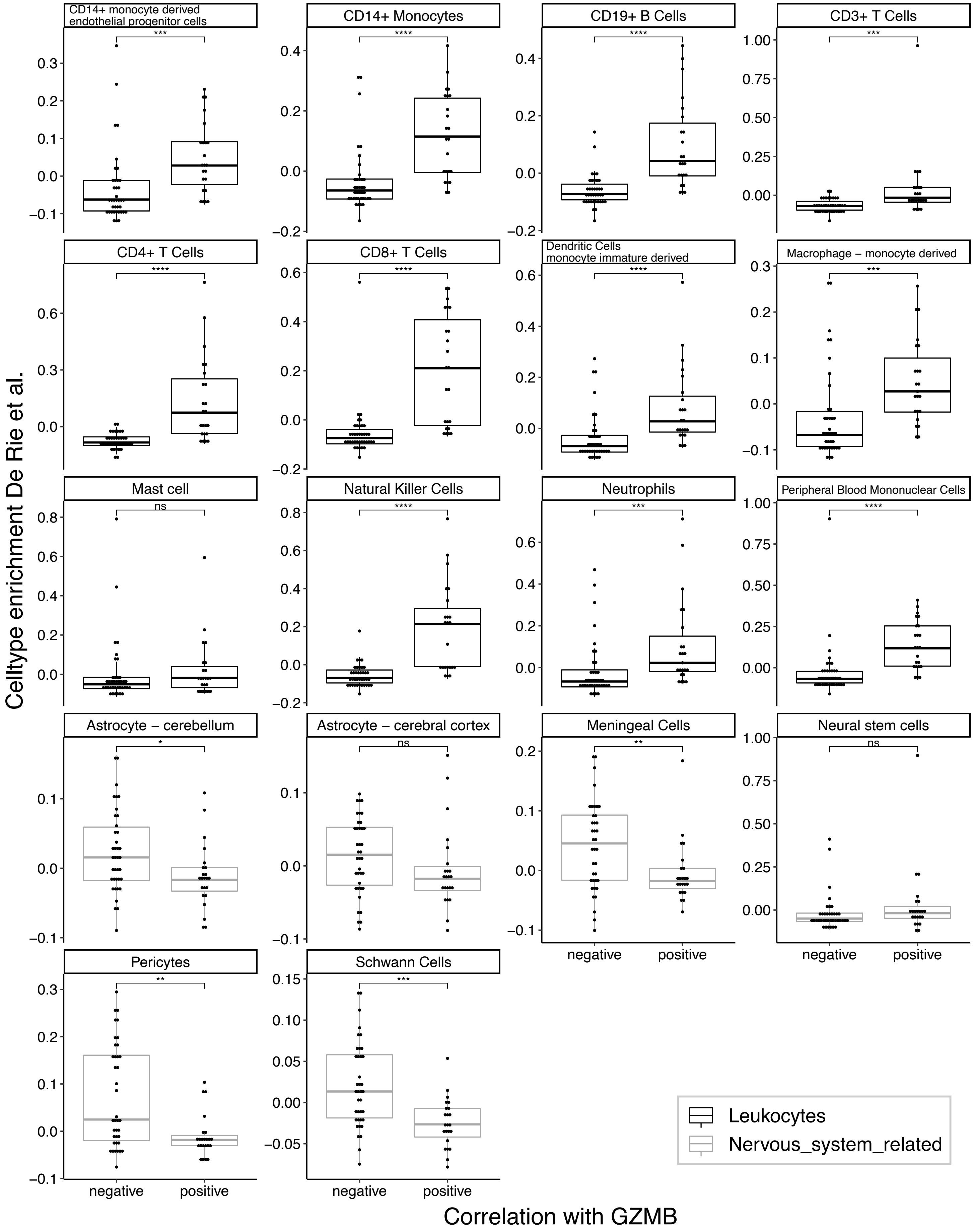

Supplementary Figure 7: miRNAs that show a positive correlation with granzyme B are enriched in immune cells. Plots show the enrichement score for the miRNA within specific celltypes as described in De Rie et al for all miRNAs that are significantly associated with Granzyme B (FDR <0.1) within the samples of the individuals that converted from relapsing-remitting to secondary-progressive MS during the study. Every datapoint represents the correlation coefficient per miRNA. P-values (Mann-Whitney U) were adjusted for FDR (ns: p > 0.05 *: p <= 0.05 **: p <= 0.01 ***: p <= 0.001 ****: p <= 0.0001).

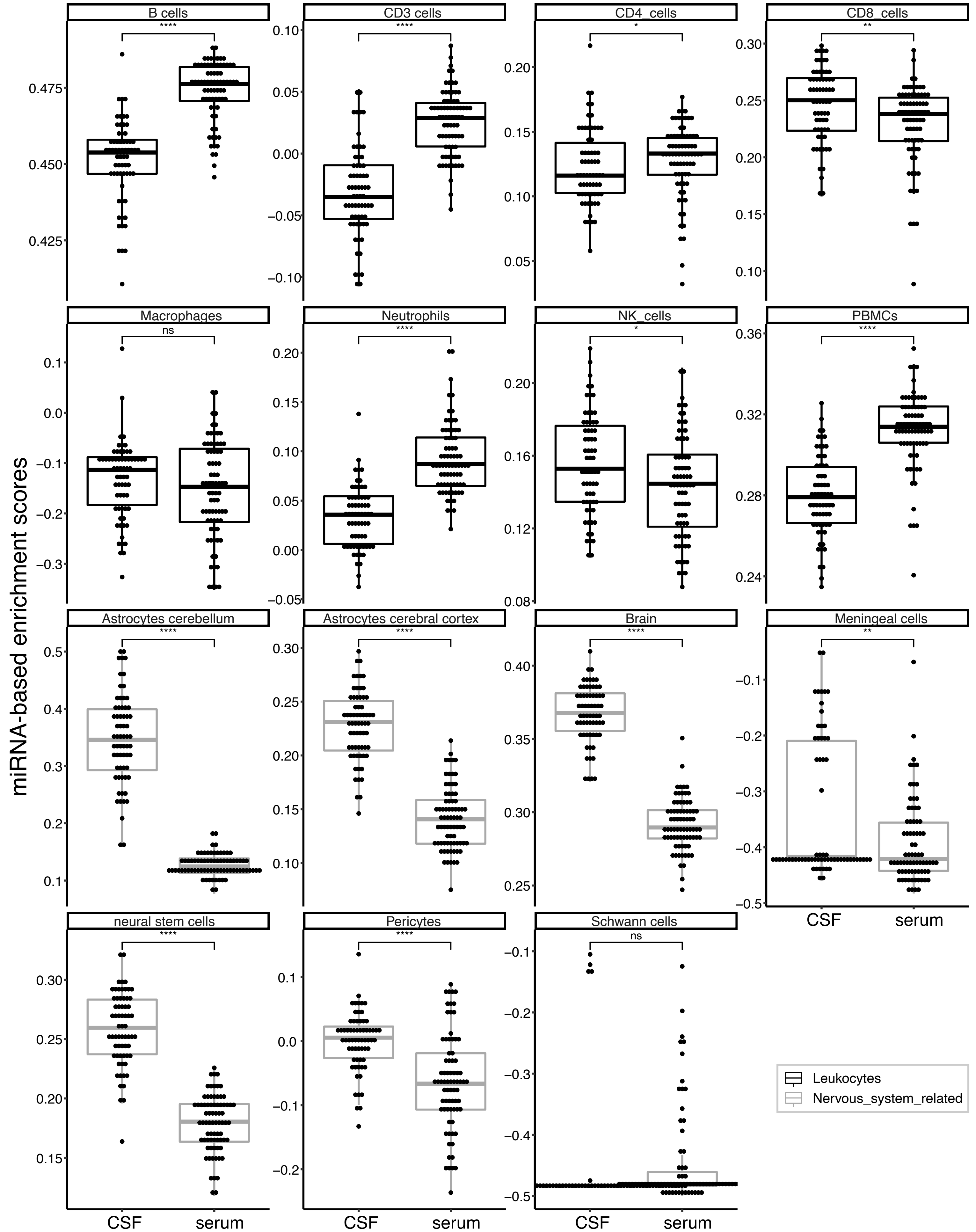

Supplementary Figure 8: miRNA-based enrichment scores for nervous tissue specific cells are enriched in cerebrospinal fluid samples versus serum samples in cohort I. Single sample enrichment scores were calculated per miRNA profile, taking the top 10 miRNAs enriched per celltype (according to De Rie et al.). For the category "Brain", we used data from Boudreau et al (2014) taking the top 20 miRNAs expressed in post-mortem brain samples. Every datapoint represents one sample. P-values (Mann-Whitney U) were adjusted for FDR (ns: p > 0.05 *: p <= 0.05 **: p <= 0.01 ***: p <= 0.001 ****: p <= 0.0001).

Supplementary methods

Supplementary Methods: Additional information about quality control of microRNA data.
Supplementary Figure 9: Library size (after alignment) versus the diversity of the library (number of different microRNAs that have >1 count after alignment) in cohort I (CSF and serum) and cohort II (serum).
Supplementary Figure 10: Principal component analyses in cohort I in different quality control steps.
Supplementary Figure 11: Principal component analyses in cohort II in after exclusion of samples in quality control step 1.

Supplementary Methods: Additional information about statistical methods.

Supplementary methods: Additional information about quality control of microRNA data

We included only high-quality microRNA (miRNA) sequencing data in the final differential expression analyses. In quality control (QC) step 1, we excluded samples with low library diversity and/or low total library size. For all serum samples, we used the following cut-offs: 100 different aligned miRNAs and 1000 total library size (see Supplementary Figure 9). For the CSF samples, we used a lower cut-off for diversity (50 different miRNAs) because CSF samples are known to harbor fewer miRNAs (see Supplementary Figure 9*)*. In cohort I (cross-sectional case-control cohort), the low quality samples that were excluded in QC step 1 cluster together in a principal component analysis (PCA), and this clustering was independent of the technical batch (see Supplementary Figure 10A/B). In cohort II, only one sample was excluded in QC step 1.

Next, we recalculated the PCA after exclusion of the QC step 1 samples and excluded the major outliers in the PCAs (looking at PCs 1–4) (Supplementary Figure 10C/D and Supplementary Figure 11). After this QC step 2, we did not observe major outliers in subsequent PCAs in the first 4 PCs, and the remaining high-quality miRNA profiles clustered based on sample type (see Fig. 5 in the main manuscript).

The miRNA libraries of cohort II yielded more miRNA reads (library size $1.1*10^6$ [IQR $0.8–1.4*10^6$]) and were richer (diversity 569 [IQR 504–617) than those of cohort I (library size $1.7*10^4$[IQR $0.7–4.1*10^4$]; diversity 332 [IQR 240–415]) (Supplementary Figure 9*,* Supplementary Table 1). This higher library size and diversity also resulted in more miRNAs being reliably detected (>5 raw counts on average) in cohort II. miRNAs that could be detected in cohort II but not in cohort I were relatively lowly expressed miRNAs in cohort II, with average raw read counts ranging from 5 to 45 raw reads. This indicates that, while more "rare" miRNAs could be detected in cohort II, the highly abundant miRNAs in serum overlap between both cohorts.

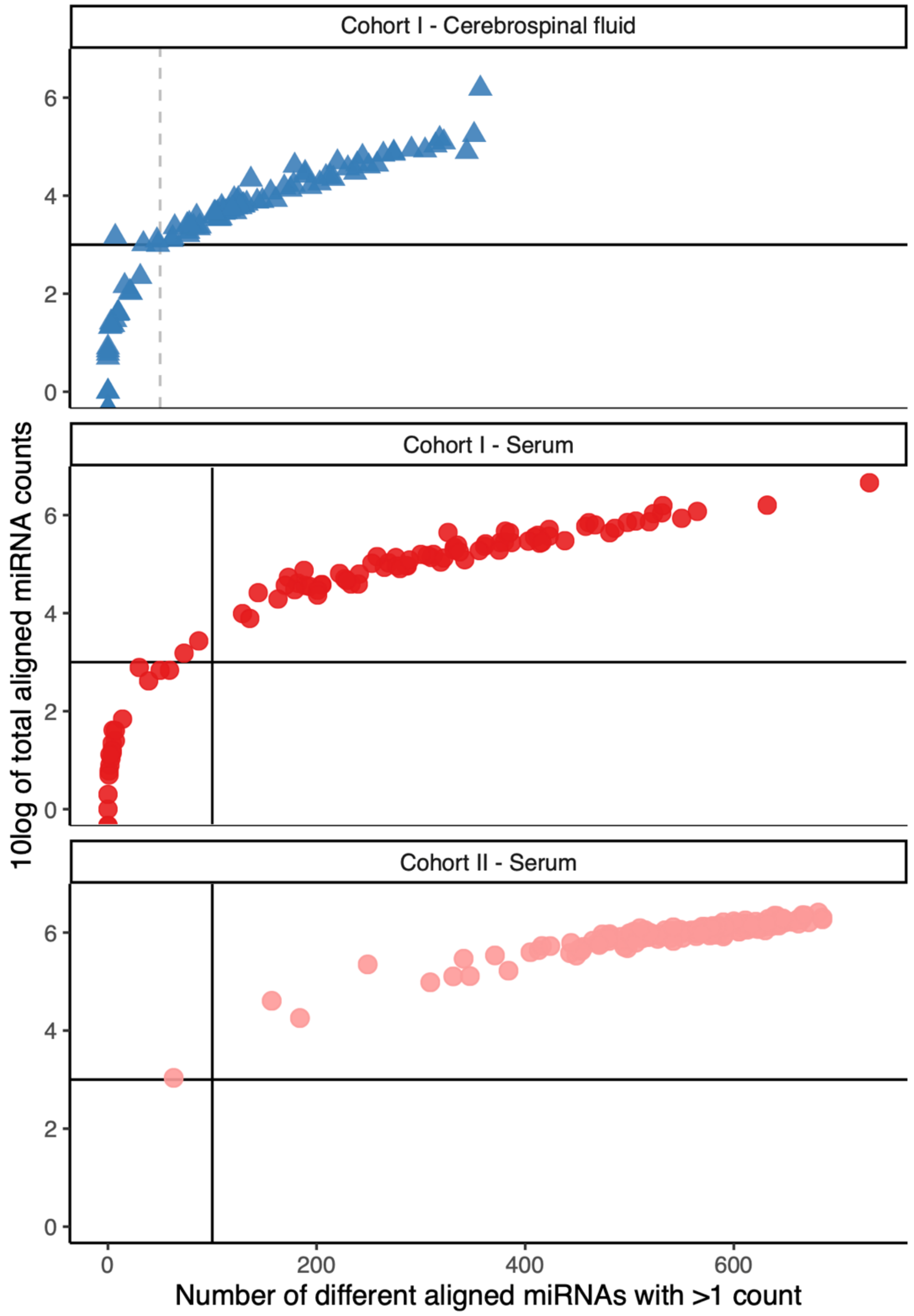

Supplementary Figure 9: Library size (after alignment) versus the diversity of the library (number of different microRNAs with >1 count after alignment) in cohort I (CSF and serum) and cohort II (serum). Horizontal lines mark the cut-off for library size (1000 total aligned miRNA reads). Vertical lines mark the cut-off for diversity (gray dotted line is the cut-off for the CSF samples: 50 different aligned miRNAs, black line is the cut-off for the serum samples: 100 different aligned miRNAs).

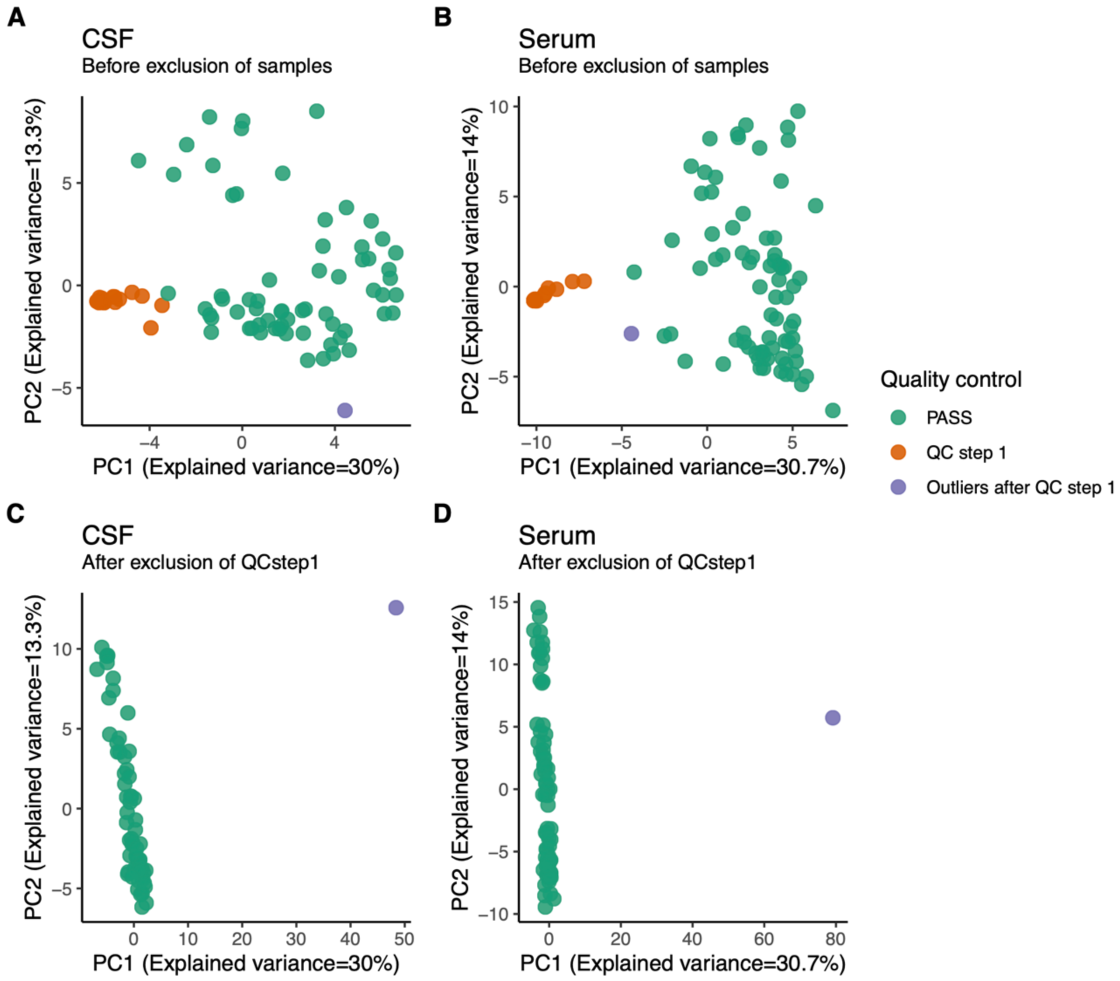

Supplementary Figure 10: Principal component analyses in cohort I in different QC steps. Samples were excluded in QC step 1 based on library size (<1000 aligned miRNA counts) and/or diversity (<50 (CSF) or <100 (serum) different miRNAs detected). The samples that are excluded in QC step 1 cluster together both in **A)** CSF as well as **B)** serum. After exclusion of samples in QC step 1, additional clear outliers were removed based on the PCs of **C)** CSF and **D)** serum.

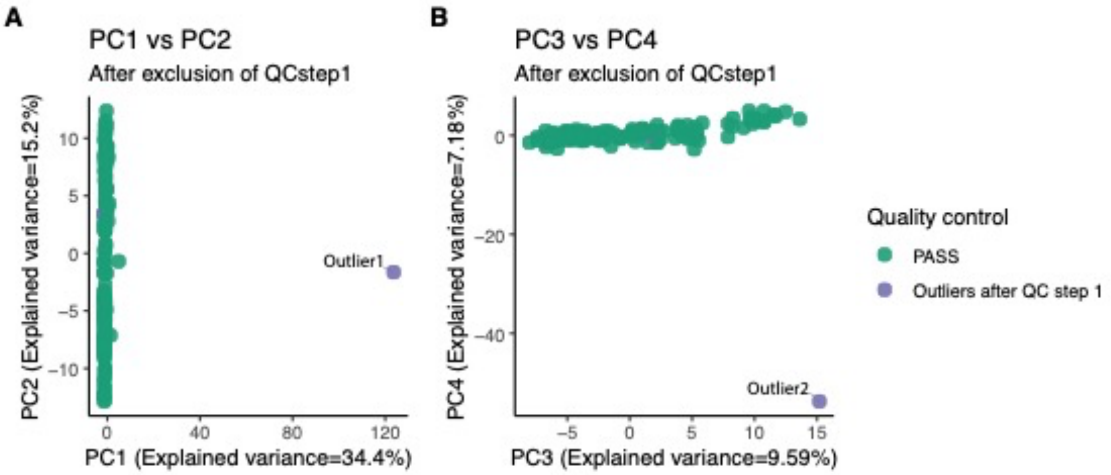

Supplementary Figure 11: Principal component analyses in cohort II in after exclusion of samples in QC step 1. The PCA were performed after exclusion of one sample in QC step 1 (<1000 aligned miRNA counts and/or <100 different miRNAs detected). Two additional samples were removed that were clear outliers in PC1/2 (Outlier1) or PC3/4 (Outlier2).

Supplementary methods: Additional information about the statistical methods

Baseline characteristics

Depending on the distribution of the outcome variable assessed by the Shapiro-Wilks test, either non-parametric or parametric tests were performed. Linear regression models were used to assess differences between disease subtypes while correcting for age and sex. Pearson correlation was used to correlate levels in CSF and serum for the protein levels (NpX) and miRNA levels.

Generating ROC curves for proteomics data

We tested whether the 13 proteins in CSF that differed between MS and controls can distinguish MS from controls. The concentrations of proteins were normalized by natural logarithm transformation and scaled by subtracting the mean of the value and dividing it by the standard deviation. Transformed values were used to construct multivariate generalized linear models to predict MS vs controls, and the model was optimized by backward feature selection to identify the top-performing combination of the biomarkers. Model construction, optimization and evaluation was performed using 10-fold cross-validation with the goal of optimizing the Cohen's Kappa value, and the optimized biomarker set was selected as the set that achieved 98% of the maximum Kappa value observed during the feature elimination. Model training, optimization and testing was performed using the Caret package (v.6.0-80, https://topepo.github.io/caret/).

Differential expression analysis on miRNA data

Differential expression analysis of the miRNA sequencing data was performed using the DESeq2 package, using batch, age and sex as covariates (version 1.22.2). P-values for proteins and miRNAs were corrected for the number of analytes tested according to Benjamini-Hochberg (adjusted p-value (FDR))[1]. miRNAs were considered significantly differentially expressed at an FDR < 0.1. PCA was performed to identify outliers in the data (uncorrected regularized log normalized counts) and to assess differences in global miRNA counts between diagnostic groups (corrected for sex, batch and age). For visualization purposes, miRNAs were normalized with DESeq2 using the regularized log function and corrected for batch, age and sex (limma version 3.42.2).

Meta-analysis

Before this meta-analysis, a Cochrane's Q test was performed and, for all analytes that did not show significant heterogeneity (Cochrane's Q P-value > 0.05), a fixed-effects meta-analysis was performed using the inverse-variance method to pool the log2fold changes and the standard errors of different comparisons (meta package, version 4.9-5, https://github.com/guido-s/meta/ https://link.springer.com/book/10.1007/978-3-319-21416-0).

Supplementary reference

1. Benjamini, Yoav ; Hochberg Y. <Benjamini&Hochberg1995_FDR.pdf>. *J R Stat Soc Ser B* 1995; 57: 289–300.
